# R3HDM4 influences kidney renal clear cell carcinoma progression, immune modulation, and potential links to the IGSF8 immune checkpoint

**DOI:** 10.3389/fimmu.2025.1722358

**Published:** 2025-11-19

**Authors:** Kai Sun, Rong Li, Ting Xu, Song Wen, De-chang Xu, Ke-run Wang

**Affiliations:** 1 Department of Oncology, Ganzhou Cancer Hospital, The Affiliated Cancer Hospital of Gannan Medical University, Ganzhou, Jiangxi, China; 2 Department of Pathology, Ganzhou Cancer Hospital, The Affiliated Cancer Hospital of Gannan Medical University, Ganzhou, Jiangxi, China

**Keywords:** R3HDM4, renal clear cell carcinoma, prognosis, immune infiltration, IGSF8

## Abstract

**Background:**

R3HDM4, or R3H domain containing 4, is a gene with uncertain functions but is frequently investigated for its potential cellular roles and associations with various diseases. Kidney renal clear cell carcinoma (KIRC ), a prevalent and aggressive form of kidney cancer, currently lacks effective treatment options. This study aimed to clarify the involvement of R3HDM4 in KIRC pathogenesis.

**Methods and results:**

An integrated pan-cancer approach was employed to analyze data from The Cancer Genome Atlas (TCGA), Gene Expression Omnibus (GEO), ArrayExpress, and the International Cancer Genome Consortium (ICGC ), systematically assessing the prognostic relevance, clinical associations, signaling pathways, DNA methylation patterns, immune infiltration profiles, and chemotherapeutic sensitivity linked to R3HDM4 expression. Bioinformatics analyses, supported by immunohistochemistry, Western blotting (WB), and reverse transcription-quantitative polymerase chain reaction (RT-qPCR), revealed significant upregulation of R3HDM4 in KIRC tissues compared to normal controls. Kaplan–Meier (KM) survival analysis indicated that elevated R3HDM4 expression correlated with poor clinical outcomes. Single-cell RNA sequencing identified cancer cells and dendritic cells as the primary sources of R3HDM4 within the KIRC tumor microenvironment. Functional assays using R3HDM4-targeting siRNA demonstrated that its depletion suppressed the proliferative, migratory, and invasive capabilities of KIRC cells. At the molecular level, R3HDM4 knockdown attenuated epithelial–mesenchymal transition (EMT), as evidenced by increased E-cadherin expression and reduced levels of vimentin and matrix metalloproteinases MMP-2 and MMP-9. Comprehensive immune profiling revealed significant correlations between R3HDM4 expression and several immunological parameters, including immune cell infiltration, immune checkpoint expression, tumor mutational burden (TMB), and microsatellite instability (MSI). Notably, silencing of R3HDM4 led to increased expression of Immunoglobulin Superfamily Member 8 (IGSF8).

**Conclusions:**

These analyses identify R3HDM4 as a critical oncogenic driver in KIRC, potentially acting through two mechanisms: promoting tumor growth and metastasis while also exerting immunomodulatory effects, possibly mediated by IGSF8. This suggests a potential role for IGSF8 in regulating immune checkpoints, though this remains speculative. These findings highlight R3HDM4’s potential as both a prognostic biomarker and a therapeutic target in KIRC.

## Introduction

KIRC, the most common kidney cancer subtype, accounts for about 75% of malignant renal tumors (1, 2). Advances in treatment, particularly with targeted therapies and immunotherapies, have significantly improved patient survival (3, 4). Targeted therapies inhibit the VEGF pathway, while immunotherapies use immune checkpoint inhibitors (ICIs) like PD-1 and CTLA-4 to boost the immune response against tumors (5, 6). Combining these approaches is now standard for advanced KIRC (7, 8). Additionally, combining ICIs with tyrosine kinase inhibitors (TKIs) shows promise in improving outcomes for patients with higher-risk disease (9–13). Despite advancements, challenges persist due to resistance to immunotherapy and side effects impacting survival. Researchers are exploring new strategies, like identifying biomarkers and targeted therapies, to improve personalized treatment (14, 15). For instance, EMX2 has shown potential in inhibiting cholangiocarcinoma by affecting the Akt/FOXO3a pathway (16). KIRC molecular heterogeneity and conventional therapy resistance prompted research on alternative targets such as BUB1B, a key KIRC progression driver from genome-wide transcriptomic analyses (17). BUB1B inhibitors elicit apoptosis and hold therapeutic potential. Despite advances in KIRC treatment, further research is critical to address drug resistance and refine therapeutic strategies. Combining targeted and immunotherapies with novel biomarkers and therapeutic targets discovery offers substantial promise for improving KIRC treatment outcomes (3, 18).

R3HDM4, also known as C19orf22 and formally designated as R3H domain containing 4, is located on the short arm (p) of chromosome 19 at region 13.3. This gene encodes the protein MGC16353, listed in genomic databases as Ensemble ID ENSG00000198858 and UniProt Q96D70. The R3HDM4 protein features an R3H domain, which is typically associated with RNA regulation in cells (19, 20). The significance of the R3H domain is highlighted by its presence in various proteins across multiple organisms, highlighting its evolutionary conservation and functional relevance (20, 21). This domain is characterized by its ability to bind single-stranded nucleic acids, a critical function in processes such as transcriptional regulation and RNA metabolism. R3HDM4’s role is closely linked to the modulation of RNA metabolism, influencing RNA stability, transport, and translation. Despite being frequently studied in relation to its involvement in diverse cellular processes and disease mechanisms, the specific functions of R3HDM4 remain largely undefined. Genes like R3HDM4 are often identified through genome-wide association studies (GWAS) and other genetic analyses that explore associations between genetic variants and diseases, including autoimmune and neurodegenerative disorders (22). Understanding the roles of genes like R3HDM4 and their interactions with other genetic elements is crucial for uncovering the molecular mechanisms underlying various diseases. Such insights could inform the development of targeted therapies aimed at mitigating the effects of genetic predispositions. As research progresses, the characterization of R3HDM4 and similar genes is expected to significantly enhance our understanding of human genetics and disease.

This study utilized extensive multi-omics datasets from TCGA, GEO, ArrayExpress, and ICGC to comprehensively analyze R3HDM4 expression profiles and evaluate their clinical relevance in KIRC. Techniques such as immunohistochemistry, western blotting, and quantitative PCR revealed significantly elevated R3HDM4 levels in KIRC tissues compared to normal renal samples. Integrative analyses, including DNA methylation profiling, single-cell transcriptomics, and drug sensitivity assessments, further elucidated the biological functions of R3HDM4 in KIRC pathogenesis. Cellular experiments demonstrated that silencing R3HDM4 significantly reduced the malignant characteristics of KIRC cells, such as proliferation, invasiveness, and metastatic potential, accompanied by changes in epithelial–mesenchymal transition (EMT) regulators (E-cadherin and vimentin) and extracellular matrix remodeling enzymes (MMP-2 and MMP-9). Furthermore, this study conducted a systematic investigation into the impact of R3HDM4 on the immunological landscape of KIRC tumors. The expression of R3HDM4 was found to correlate with immune checkpoint molecules and immune cell infiltration, indicating its potential role in modulating the KIRC immune microenvironment. Overall, these findings underscore the pivotal role of R3HDM4 in KIRC tumorigenesis and offer valuable insights for the development of targeted therapeutic strategies. 

## Materials and methods

### Data collection, preprocessing, and expression analysis

Comprehensive genomic and transcriptomic datasets from various cancer subtypes were systematically downloaded from the TCGA database (https://portal.gdc.cancer.gov/). For comparative analysis with tumor samples, tumor-matched normal tissue expression profiles were obtained from the Genotype-Tissue Expression (GTEx) project (http://www.gtexportal.org/) (23). The study cohort included 28 normal kidney specimens from GTEx, 72 adjacent non-tumor tissues from KIRC cases, and 531 KIRC tumor samples along with corresponding clinical annotations from TCGA-KIRC. Detailed clinicopathological characteristics of TCGA-KIRC are presented in **Supplementary Table S1**. To further validate our findings, additional KIRC datasets were incorporated from the GEO repository (https://www.ncbi.nlm.nih.gov/geo/), including GSE167573 (63 tumor and 14 normal specimens), GSE22541 (68 tumor samples), and GSE29609 (39 tumor samples) (24). Further data were sourced from the ICGC portal (https://dcc.icgc.org/) and the E_TABM_1980 dataset (101 KIRC cases) from ArrayExpress. Transcript abundance was quantified as transcripts per million (TPM) and normalized by log_2_(TPM + 1) transformation. All computational analyses were performed using R (v4.3.0) with rigorously documented pipelines, ensuring alignment with current cancer bioinformatics standards. Missing values were imputed using the missForest R package. Stringent quality control measures were implemented, and potential outliers were identified using interquartile range (IQR) assessment. Samples exceeding Q1 - 1.5 × IQR or Q3 + 1.5 × IQR were winsorized to the nearest acceptable value. Only samples with complete transcriptomic profiles and corresponding clinical metadata were included in downstream analyses. TCGA RNA-seq data (TPM-normalized) were processed using DESeq2 (v1.34.0) and limma (v3.50.3) packages to accommodate RNA-seq count distribution properties (25). GEO microarray data were normalized using the robust multi-array average (RMA) algorithm to correct for platform-specific artifacts. Differential expression analysis was conducted using consistent statistical thresholds (adjusted *P* < 0.05, |log_2_FC| > 1) across all datasets.

### Tissue specimen collection and immunohistochemical analysis

Sixteen matched pairs of KIRC samples and adjacent normal kidney tissues were obtained from Ganzhou Cancer Hospital. The study protocol was approved by the Institutional Ethics Committee (Approval Number: 2025Kelunshen236). All specimens underwent thorough histopathological verification to confirm KIRC diagnosis. Detailed clinicopathological information is provided in **Supplementary Table S2**. Selection criteria included (1): histologically confirmed KIRC cases and (2) complete clinical documentation. Exclusion criteria encompassed: (1) ambiguous pathological results, (2) missing clinical data, and (3) prior extensive systemic treatments. For immunohistochemical evaluation, tissue samples were fixed in 10% neutral buffered formalin, processed into paraffin blocks, and sectioned at 4 μm thickness. After deparaffinization and rehydration , antigen retrieval was performed using diluted citrate buffer (1:100; Boster Biological Technology, China). Sections were treated with HRP-labeled secondary antibodies (ZSGB-Bio, China), visualized with DAB substrate, and counterstained with hematoxylin. Digital image quantification was performed using Image-Pro Plus 6.0 software (Media Cybernetics, USA), with integrated optical density (IOD) values calculated from multiple high-magnification fields per sample.

### Prognosis analysis of R3HDM4

To assess patient outcomes, comprehensive survival analyses were performed using the Kaplan-Meier method, comparing key clinical endpoints— overall survival (OS), progression-free survival (PFS), disease-free survival (DFS), and disease-specific survival (DSS)—between groups with high and low R3HDM4 expression, based on median expression levels. These analyses were performed using the survival package (v3.3-1). Statistical significance of survival differences was determined *via* log-rank testing, with a significance threshold of *P* < 0.05. Survival curves, along with 95% confidence intervals and median survival estimates for each subgroup, were visualized using the survminer package (v0.4.9). Prognostic accuracy was further assessed through time-dependent receiver operating characteristic (ROC) analysis, implemented with the “timeROC” package, to estimate survival probabilities at 1-, 3-, and 5-year intervals. Corresponding ROC curves and area under the curve (AUC) values were derived (26). To validate the R3HDM4 expression trends in KIRC, external validation was performed using independent datasets from GEO and ICGC (24). Additionally, univariate and multivariate Cox regression models were applied to comprehensively explore potential prognostic factors.

### Functional annotation analysis of R3HDM4 in KIRC

To investigate the functional role of R3HDM4 in KIRC, systematic functional annotation was conducted *via* Gene Ontology (GO) and Kyoto Encyclopedia of Genes and Genomes (KEGG) pathway enrichment analyses (27). The GO method, a widely used approach in functional genomics, facilitated an in-depth examination of R3HDM4-related biological processes, molecular functions, and cellular localization patterns in KIRC (28). For a more comprehensive pathway-level analysis, Gene Set Enrichment Analysis (GSEA) was performed, a robust computational tool that identifies coordinated expression changes in functionally related gene clusters across diverse biological contexts (29). All computational procedures were carried out using advanced bioinformatics tools: the ClusterProfiler package (v3.14.3) in R was employed for GO and KEGG analyses, while GSEA (v4.1.0) was utilized for pathway enrichment evaluation. Protein–protein interaction (PPI) networks were constructed using the STRING database (v9.1), which enabled the identification of potential molecular interactions among co-expressed genes (30). Network visualization and analysis were performed using the GeneMANIA plugin in Cytoscape, integrating interaction data from multiple public repositories based on the target genes, including their functional annotations. Differential expression analysis employed consistent statistical criteria (adjusted *P* < 0.05, |log2FC| > 1) across all datasets. The entire analytical workflow adhered to standardized protocols to ensure methodological rigor and statistical reliability in omics data interpretation. A false discovery rate (FDR) threshold of 0.05 was applied to ensure result robustness.

### DNA methylation analysis

The EWAS Data Hub (https://ngdc.cncb.ac.cn/ewas/datahub/index) is a comprehensive repository for epigenome-wide association studies, consolidating DNA methylation data from 115,852 biological samples across 528 distinct diseases (31). The Shiny Methylation Analysis Resource Tool (SMART; http://www.bioinfo-zs.com/smartapp/) provides a unified computational platform for analyzing Infinium Human Methylation 450K array datasets, RNA-seq profiles, and clinical annotations across 33 TCGA-derived cancer types (32). These platforms were utilized to systematically investigate the epigenetic regulation of R3HDM4 in KIRC. Specifically, this study assessed the relationship between R3HDM4 promoter methylation levels and its transcriptional activity, clinicopathological features, and OS. Illumina HumanMethylation450K array data were preprocessed using the ChAMP package (v2.22.0), which included quality filtering (removing probes with detection *P*-values > 0.01 and cross-hybridizing probes) and normalization *via* the BMIQ algorithm. Methylation levels were quantified using beta-values (range: 0–1), with hypermethylation defined as beta > 0.6 and hypomethylation as beta < 0.2.

### Single-cell expression analysis

Transcriptomic profiling at single-cell resolution was conducted using sequencing data in.h5 format files, with comprehensive cell-type annotations sourced from the TISCH database (33). Data processing and analytical workflows were implemented *via* the MAESTRO platform and Seurat software package (v4.1.0) in R to ensure stringent quality control during preprocessing. Cellular heterogeneity was explored using t-distributed stochastic neighbor embedding (t-SNE) for dimensionality reduction and segregation of populations. The SCP1288 dataset, linked to PMID: 33711272, included 8 clinical samples, 3 from patients not receiving immune checkpoint blockade (ICB) treatment and 5 from patients treated with ICB and other therapies (34). This dataset underwent normalization, detection of highly variable features, and unsupervised clustering to identify distinct cell subpopulations. Rigorous quality control was maintained throughout the analysis. The preprocessing pipeline included (1): filtering out low-quality cellular profiles (retaining cells expressing 200–2000 genes and excluding those with > 5% mitochondrial gene content) (2), normalization using the LogNormalize method, and (3) feature scaling. Cellular clustering was performed using the Louvain community detection algorithm (cluster resolution parameter optimized to 0.5 for biological interpretability), followed by cell-type annotation based on established markers (hepatocytes: AFP; T lymphocytes: CD3D). Spatial distribution patterns of R3HDM4 expression were examined using UMAP projections, and expression levels across clusters were visualized with violin plots.

### Correlation analysis of immune-related indices and R3HDM4 in KIRC

To elucidate the relationship between R3HDM4 expression and tumor microenvironment characteristics in KIRC, a comprehensive immunogenomic analysis was conducted using multi-omics data from TCGA, GEO, ICGC, and ArrayExpress databases. The analytical framework incorporated seven advanced immune deconvolution algorithms (ssGSEA, xCell, CIBERSORT, EPIC, TIMER, MCP-counter, and quanTIseq), implemented through R packages such as immunedeconv, estimate, and GSVA. This multi-method approach enabled a systematic evaluation of immune cell composition, stromal content, immune activity indices, and genomic instability parameters (TMB and MSI). Additionally, co-expression patterns between R3HDM4 and 150 immunomodulatory genes across five key immune pathways were examined: (1) chemotactic signaling (41 genes), (2) immune checkpoint regulation (18 genes), (3) antigen presentation machinery (21 genes), (4) immunosuppressive mediators (24 genes), and (5) immunostimulatory factors (46 genes) (35). All computational analyses were performed using R version 4.3.0, with data visualization generated through ggplot2, pheatmap, and ggstatsplot packages to ensure rigorous statistical interpretation and accurate graphical representation.

### Drug sensitivity of R3HDM4 in KIRC

Comprehensive drug sensitivity data were obtained from three established public resources: the Cancer Therapeutics Response Portal (CTRP v2.0), the PRISM Repurposing dataset, and the Genomics of Drug Sensitivity in Cancer (GDSC) database. To explore potential correlations between R3HDM4 expression and drug efficacy, Spearman's rank correlation tests were conducted on 217 therapeutic compounds , including kinase inhibitors, epigenetic regulators, and conventional chemotherapy agents. All analyses were performed in R (v4.3.0), using the tidyverse package suite for data manipulation, the pRRophetic package for predictive drug response modeling, and ComplexHeatmap for comprehensive graphical representation of the results (36).

### Cell culture

HEK-293K (normal human kidney cell line) and 786-O (KIRC cell line) were obtained from Sangon Biotech (Shanghai, China). 786-O cells were cultured in RPMI-1640 medium (Procell, Cat. No. PM150110) supplemented with 10% fetal bovine serum (FBS, Gibco, Grand Island, NY, USA) and 1% penicillin/streptomycin (Solarbio, Beijing, China) in a humidified incubator at 37˚C with 5% CO_2_.

### siRNA-mediated R3HDM4 knockdown

Twenty-four hours prior to transfection, 786-O cells were seeded in 6-well plates and cultured to 80-90% confluence. Transfection was carried out using Lipofectamine reagent (KeyGEN, China) following the manufacturer’s instructions, with either R3HDM4-targeting siRNA or non-targeting control siRNA. Cells were harvested 24 hours post-transfection for RNA and protein extraction. Six experimental groups were included: untreated 786-O cells (CTRL), negative control siRNA (siNC), and four R3HDM4-specific siRNAs: si-R3HDM4-1 (si-R3HDM4-202), si-R3HDM4-2 (si-R3HDM4-391), si-R3HDM4-3 (si-R3HDM4-562), and si-R3HDM4-4 (si-R3HDM4-788). Preliminary screening indicated that si-R3HDM4–788 achieved the highest silencing efficiency and was selected for subsequent functional assays.

The sense and antisense sequences of the siRNAs used were as follows:

si-R3HDM4-202: sense: 5'- AACAGCACUUCAUCAACCATT-3'; antisense: 5'- UGGUUGAUGAAGUGCUGUUTT-3'.

si-R3HDM4-391: sense: 5'- GCAACAACGCCACCUAUGUTT-3'; antisense: 5'- ACAUAGGUGGCGUUGUUGCTT-3'.

si-R3HDM4-562: sense: 5'- AGUGCUUCCAGCGCAUCAGTT-3'; antisense: 5'- CUGAUGCGCUGGAAGCACUTT-3'.

si-R3HDM4-788: sense: 5'- GCAGAUGAAGGUCAGUAAUTT-3'; antisense: 5'- AUUACUGACCUUCAUCUGCTT-3'.

The sense and antisense sequences of negative control siRNA (siNC) were as follows:

siNC: sense: 5'- UUCUCCGAACGUGUCACGUTT-3'; antisense: 5'- ACGUGACACGUUCGGAGAATT-3'.

### RT-qPCR assay

Total RNA was extracted using RNA Isolater Total RNA Extraction Reagent (VAZYME) following the manufacturer’s protocol. The purified RNA was reverse-transcribed into complementary DNA (cDNA) using HiScript® II Q RT SuperMix for qPCR (+gDNA wiper) (VAZYME). Quantitative real-time PCR (qPCR) was performed on the synthesized cDNA using ChamQ SYBR qPCR Master Mix (VAZYME). Relative gene expression levels were calculated using the comparative threshold cycle (2^-ΔΔCt^) method. The specific primer sequences used in this study were as follows:

R3HDM4: forward, 5'- CACCCAGTACCTCCTGACCC -3';

reverse, 5'- GAAATCGTTCCAGACCTCCAC -3', 142 bp.

GAPDH: forward, 5'- ATGGGGAAGGTGAAGGTCGGAGT -3';

reverse, 5'- TAGTTGAGGTCAATGAAGGGGTC -3', 125 bp.

### Western blot for detecting protein expression

Following transfection, cells were washed twice with PBS and lysed in ice-cold RIPA buffer. Protein concentrations were quantified using a BCA assay kit (GBCBIO, China). Electrophoresis was performed on 10% SDS-PAGE gels, followed by transfer to nitrocellulose membranes (Biofroxx, Germany). Membranes were blocked with 5% skim milk for 2 hours at room temperature before overnight incubation with primary antibodies at 4°C. Between each incubation step, membranes were washed three times for 10 minutes with TBST. HRP-conjugated goat anti-mouse IgG (1:10,000, Boster, China) was used as the secondary antibody, with identical washing conditions applied prior to chemiluminescent detection. The primary antibodies used were: R3HDM4 (29 kDa, 1:1000, Invitrogen, USA), GAPDH (36 kDa, 1:10,000, Proteintech, China), E-cadherin (125 kDa, 1:40,000, Proteintech, China), Vimentin (55 kDa, 1:40,000, Proteintech, China), MMP2 (63 kDa, 1:1000, BIOSS, China), MMP9 (78 kDa, 1:1000, Affinity, USA), and IGSF8 (70 kDa, 1:2000, Proteintech, China).

### Evaluation of cell proliferation

Cell proliferation was assessed using the CCK-8 assay kit (HYCEZMBIO, China). Following transfection , cells were seeded in 96-well plates at a density of 3 × 10^3^ cells per well. Cellular viability was measured at 0, 24, and 48 hours after plating by adding 10 μL of CCK-8 solution to each well °C and incubating for 1 hour at 37°C with 5% CO_2_. Optical density at 450 nm was recorded using a microplate reader (Thermo Scientific, USA).

### Transwell assays for cell migration and invasion

Cell migration and invasion assays were performed using 24-well Transwell chambers (Corning, USA) with 8 μm pore membranes, pre-coated with 100 μL Matrigel basement membrane matrix (Corning, USA). Transfected 768-O cells (6 × 10^4^ cells per well) were seeded in serum-free medium in the upper compartment, while the lower chamber contained 600 μL complete medium supplemented with 20% fetal bovine serum as a chemoattractant. After 24-hour incubation at 37 °C, cells that migrated through the membrane were fixed with 4% paraformaldehyde for 1 hour and stained with 0.5% crystal violet for 20 minutes. The number of migrated cells was quantified by counting stained cells in five randomly selected fields per membrane using bright-field microscopy.

### Statistical analysis

Data analysis was performed using the R statistical computing environment (v4.3.0), employing multiple analytical methods. Fold-change metrics and hazard ratios (HR ) were calculated for quantitative assessment, with statistical significance determined *via* Log-rank testing. Correlation analyses utilized Spearman's rank correlation and Pearson's correlation methods. Group comparisons were made using Wilcoxon rank-sum tests, Student's t-tests (for two-group comparisons), and ANOVA (for multiple group comparisons). Survival analysis was visualized through Kaplan-Meier plots, with log-rank tests applied to assess differences, maintaining a significance threshold of *P* = 0.05. Statistical significance was represented as follows: * (*P* < 0.05), **(*P* < 0.01), ***(*P* < 0.001), and ****(*P* < 0.0001).

## Results

### Analysis of R3HDM4 expression and its correlation with clinical parameters in KIRC *via* public databases

**Figure 1:**
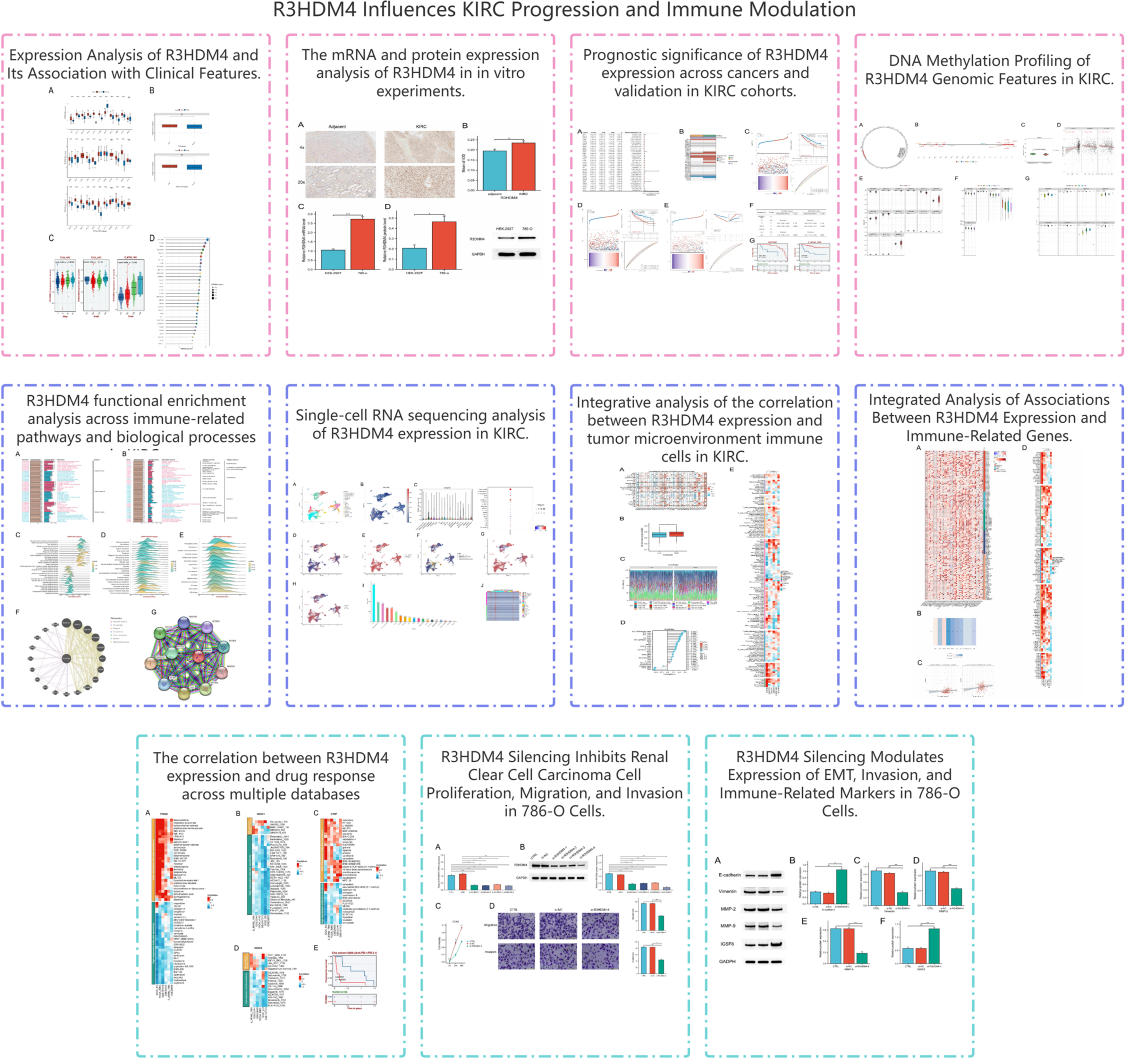
Research flowchart of this study.

**Figure 2:**
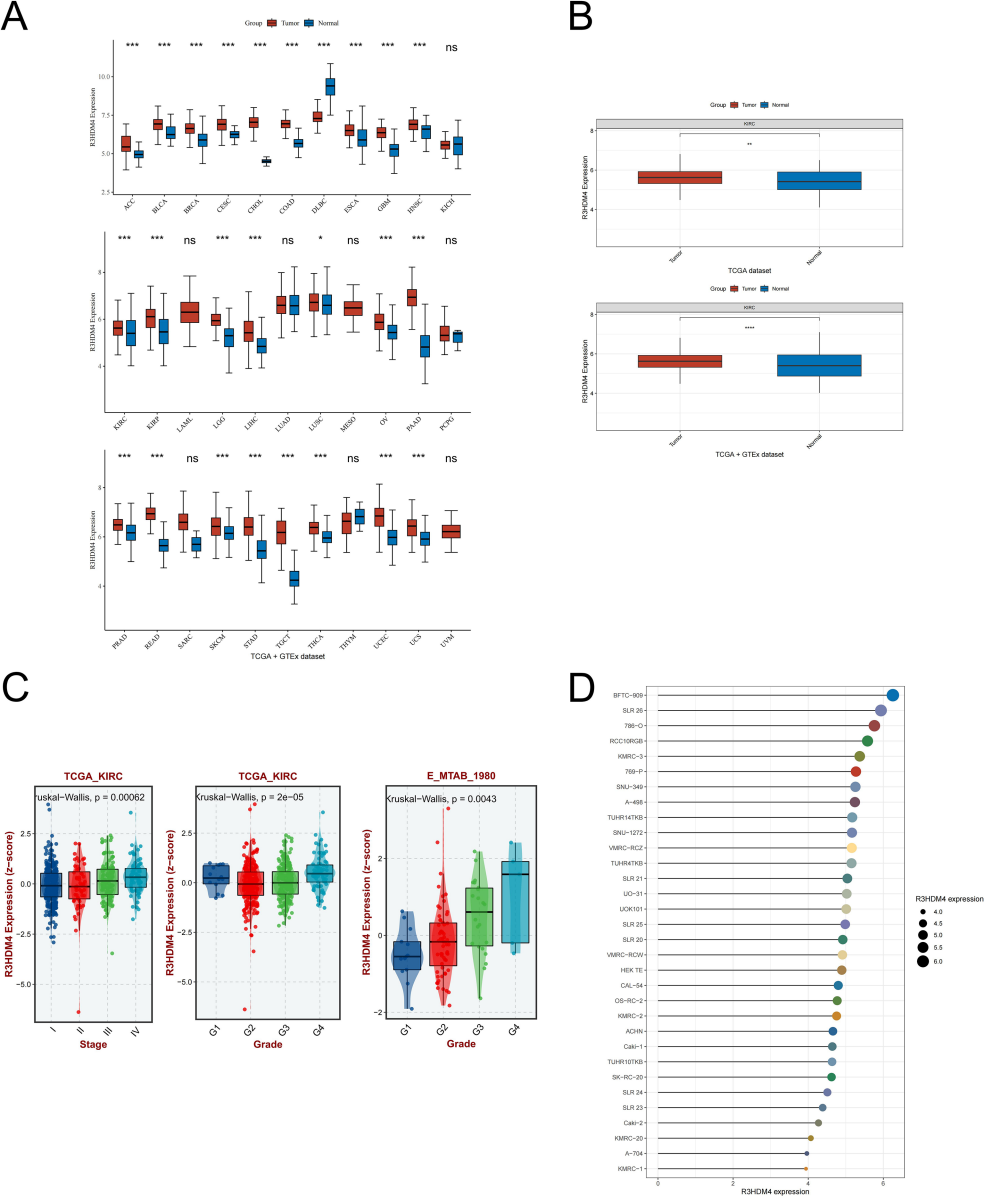
Expression analysis of R3HDM4 and its association with clinical features. **(A)** Comparative analysis of R3HDM4 expression in pan-cancer tissues versus adjacent normal tissues based on data from the TCGA and GTEx databases. **(B)** Comparative analysis of R3HDM4 expression in tumor versus normal tissues in KIRC based on data from the TCGA and TCGA + GTEx databases. **(C)** Analysis of the association between R3HDM4 expression and clinical parameters in KIRC. **(D)** Comparative analysis of R3HDM4 expression in renal clear cell carcinoma cell lines. *P < 0.05, **P < 0.01, ***P < 0.001, **** *P* < 0.0001. R3HDM4, R3H domain containing 4; KIRC, Kidney Renal Clear Cell Carcinoma; TCGA, The Cancer Genome Atlas; GEO, Gene Expression Omnibus.

**Figure 1** shows the study design flowchart. A pan-cancer analysis using TCGA and TCGA+GTEx datasets examined R3HDM4 expression across various cancers. Analysis revealed significantly higher R3HDM4 mRNA levels in tumor vs. normal tissues across multiple cancers, including ACC (adrenocortical carcinoma), BLCA (bladder urothelial carcinoma), BRCA (breast invasive carcinoma), KIRC, LIHC (liver hepatocellular carcinoma), LGG (lower grade glioma), and other common malignancies. R3HDM4 was only downregulated in diffuse large B-cell lymphoma (DLBC) (**Figure 2A**). This upregulation in multiple tumors and downregulation in DLBC highlights its context-dependent functions in different cancers, supporting further studies. In KIRC, R3HDM4 was significantly higher in tumor than normal tissues in both TCGA and combined TCGA+GTEx datasets (**Figure 2B**). Clinical analyses showed its expression correlated with advanced tumor stage (III/IV vs. I/II) in TCGA_KIRC, and with tumor grade in multiple datasets. It was higher in advanced grades in E_MTAB_1980, while in TCGA-KIRC only grade 4 was elevated compared to grades 1, 2, 3 (**Figure 2C**). R3HDM4 expression varied across renal cell carcinoma cell lines: higher in BFTC-909, SLR 26, 786-O, RCC10RGB, KMRC-3, 769-P and lower in KMRC-1, A-704, KMRC-20 (**Figure 2D**). 

### *In vitro* validation of R3HDM4 differential mRNA and protein expression

**Figure 3:**
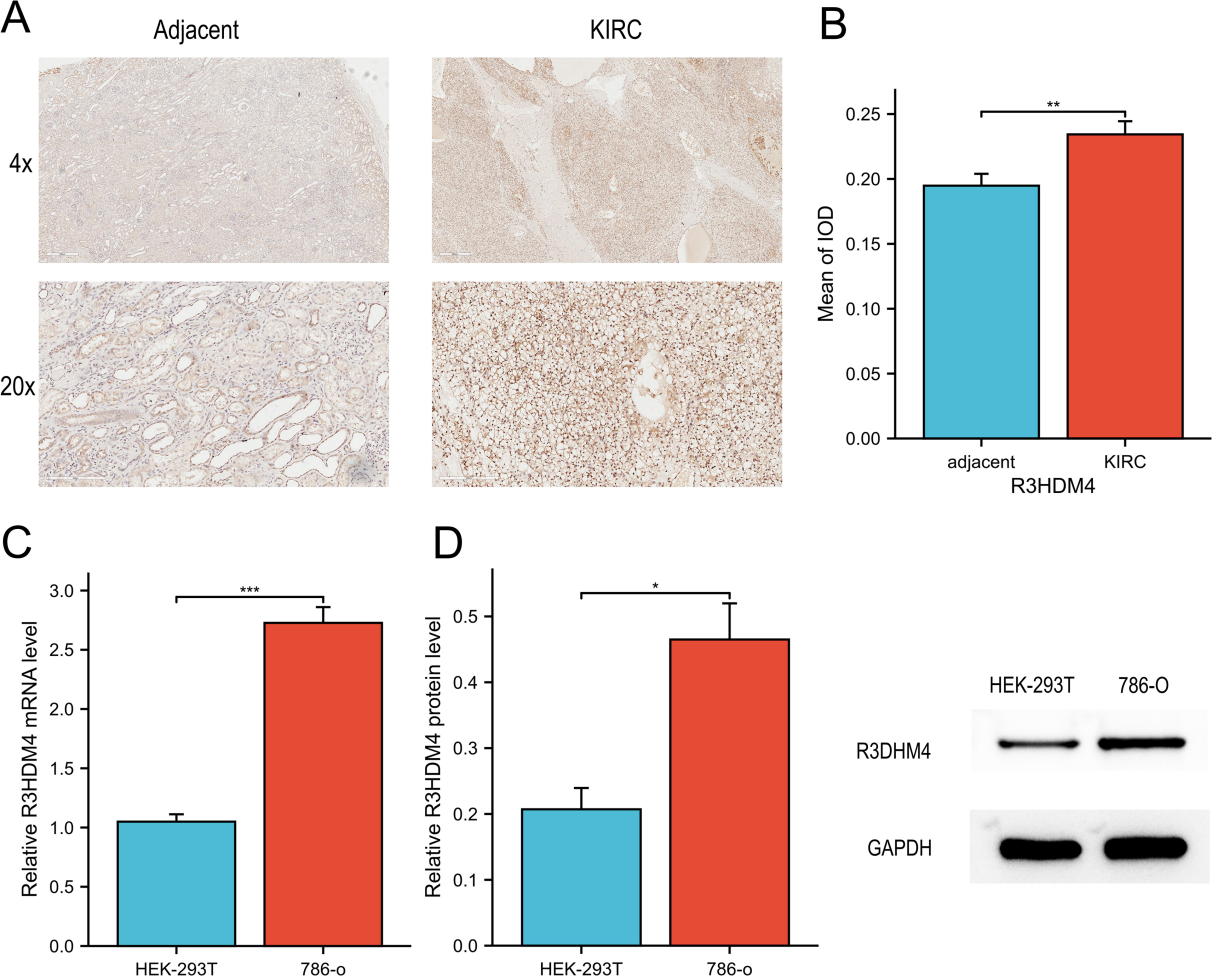
mRNA and protein expression analysis of R3HDM4 in *in vitro* experiments. **(A)** IHC analysis of R3HDM4 in KIRC tumor tissues and paired adjacent non-tumor kidney tissues. **(B)** Quantification of R3HDM4 immunostaining *via* IOD analysis. **(C)** Relative R3HDM4 mRNA levels in HEK-293K (normal kidney cell line) and 786-O (KIRC cell line) cells. **(D)** Relative R3HDM4 protein levels and representative Western blot images in HEK-293K and 786-O cells. *P < 0.05, **P < 0.01, ***P < 0.001, ****P < 0.0001. R3HDM4, R3H domain containing 4; KIRC, Kidney Renal Clear Cell Carcinoma; IHC, immunohistochemistry; IOD, integrated optical density.

To further validate R3HDM4 dysregulation in KIRC, *in vitro* assays were performed. IHC staining of 16 paired KIRC and adjacent normal tissues showed significantly stronger R3HDM4 protein staining in KIRC tissues, as seen in representative 4× and 20× images (**Figure 3A**). Mean IOD quantification confirmed elevated R3HDM4 protein levels in KIRC tissues (**Figure 3B**). qRT-PCR revealed significantly higher R3HDM4 mRNA levels in renal cancer cell line 786-O than normal renal epithelial cell line HEK-293T (**Figure 3C**). Consistent with mRNA data, Western blot demonstrated increased R3HDM4 protein in 786-O cells compared to HEK-293T cells (**Figure 3D**). Together, these results confirm R3HDM4 upregulation at both mRNA and protein levels in KIRC tissues and cells.

### Prognostic significance of R3HDM4 expression across pan-cancer, with emphasis on KIRC

**Figure 4:**
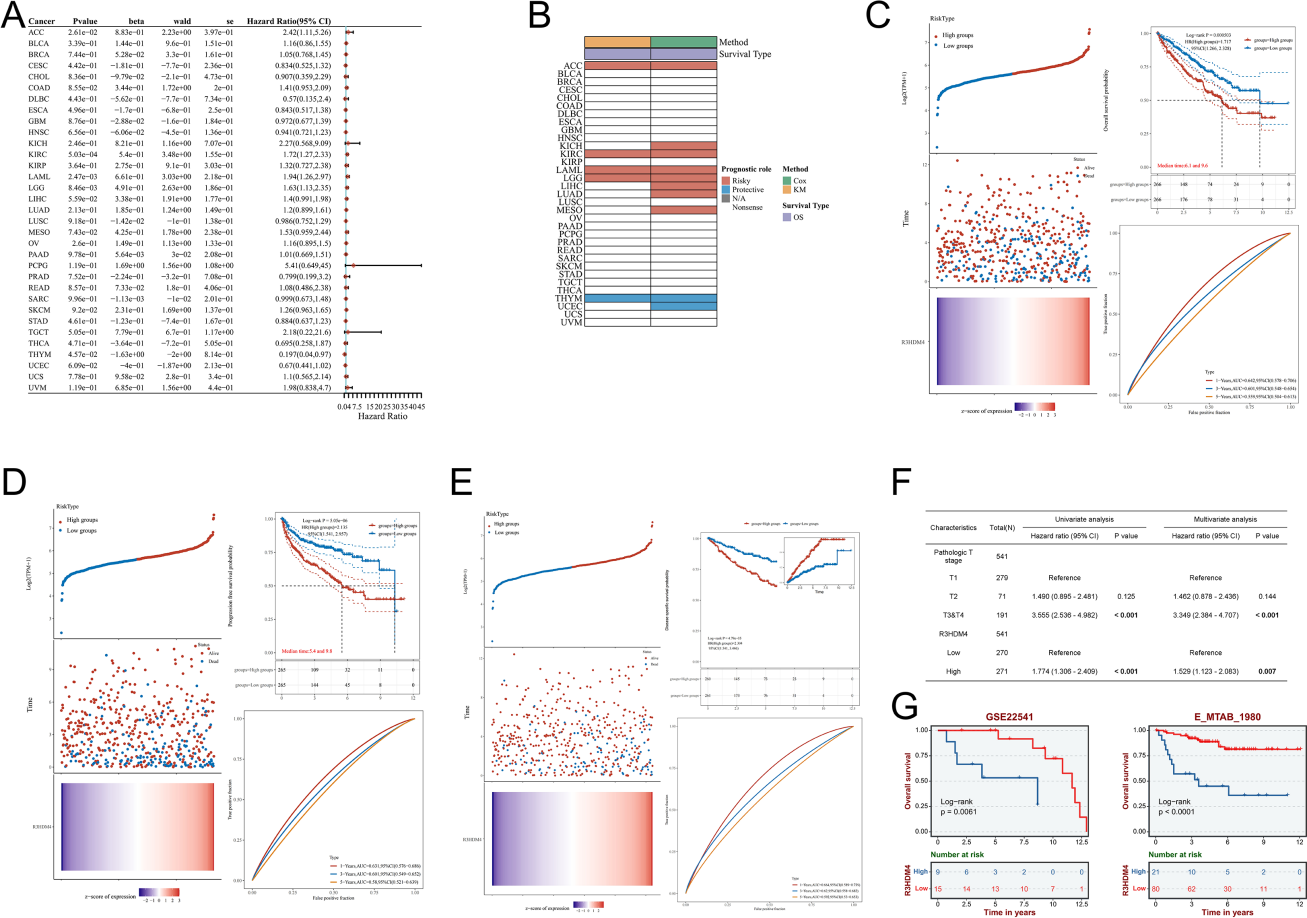
Prognostic significance of R3HDM4 expression across cancers and validation in KIRC cohorts. **(A)** Univariate Cox regression analysis of R3HDM4 expression in diverse cancer types. **(B)** Distribution of R3HDM4 expression among tumor molecular subtypes. **(C)** Prognostic analysis for OS of R3HDM4 in the TCGA-KIRC dataset. **(D)** Prognostic analysis for PFS of R3HDM4 in the TCGA-KIRC dataset. **(E)** Prognostic analysis for DSS of R3HDM4 in the TCGA-KIRC dataset. **(F)** Prognostic significance of R3HDM4 expression for PFS evaluated by univariate and multivariate analyses. **(G)** Independent validation in external GEO and ArrayExpress cohorts confirming the prognostic significance of R3HDM4 in KIRC. *P < 0.05, **P < 0.01, ***P < 0.001, ****P < 0.0001. AUC, Area Under Curve; CI, Confidence Interval; DFS, Disease-Free Survival; GEO, Gene Expression Omnibus; HR, Hazard Ratio; KIRC, Kidney Renal Clear Cell Carcinoma; OS, Overall Survival; PFS, Progression-Free Survival; RFS, Relapse-Free Survival; ROC, Receiver Operating Characteristic; TCGA, The Cancer Genome Atlas; TPM, Transcripts Per Million.

Subsequent to identifying aberrant R3HDM4 expression in KIRC, we comprehensively investigated its prognostic significance. Pan-cancer univariate Cox regression analysis for OS revealed elevated R3HDM4 expression correlated with poor prognosis in multiple malignancies, acting as a risk factor in ACC, KIRC, acute myeloid leukemia (LAML) and LGG, and a protective factor in THYM and UCS (**Figure 4A**). Kaplan-Meier (KM) analysis validated these findings, particularly for OS (**Figure 4B**). In TCGA-LIHC, high R3HDM4 expression significantly correlated with worse OS (HR = 1.717), PFS (HR = 2.135) and DSS (HR = 2.304) (**Figures 4C-E**). Time-dependent ROC analysis for KIRC demonstrated moderate predictive accuracy, with 1-year AUC values of 0.642 for OS, 0.631 for PFS and 0.664 for DSS, and slight decreases at 3 and 5 years (**Figures 4C-E**). Univariate analysis showed high R3HDM4 expression significantly increased OS risk, a finding further validated by multivariate analysis. Pathologic T stage (T3/T4 vs. T1/T2) also emerged as an independent prognostic factor (**Figure 4F**). External validation in independent datasets (E_MTAB_1980, GSE22541) confirmed R3HDM4’s adverse prognostic role, with high expression associating with reduced OS as depicted in KM curves (**Figure 4G**).

### DNA methylation analysis of R3HDM4 in patients with KIRC

**Figure 5:**
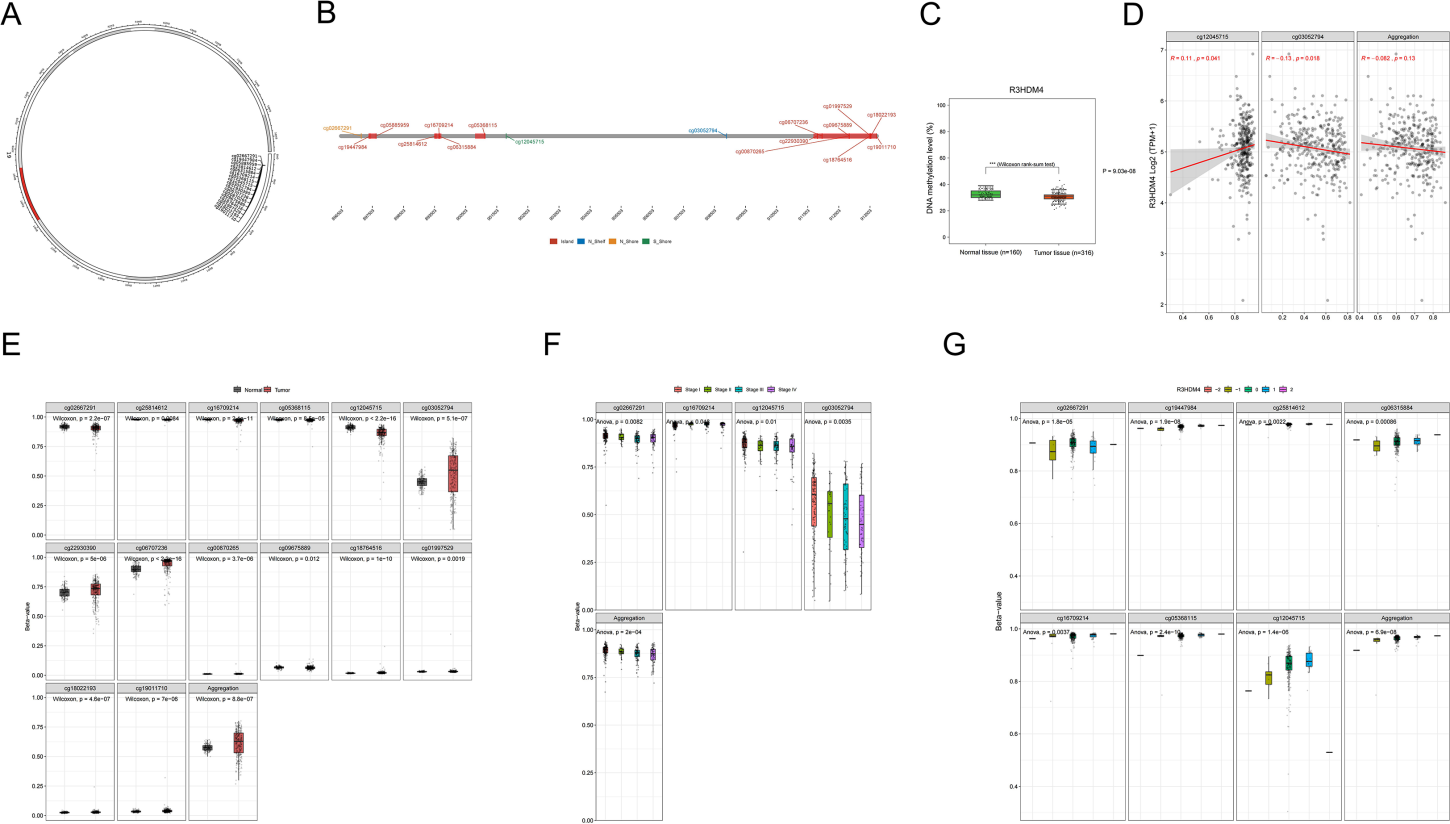
DNA methylation profiling of R3HDM4 genomic features in KIRC. **(A)** Chromosomal localization of R3HDM4 in the human genome. **(B)** Genomic architecture of R3HDM4 and its flanking regions. **(C)** Dynamics of promoter methylation in KIRC versus normal kidney tissues. **(D)** Analysis of the correlation between R3HDM4 expression and its methylation status. **(E)** Comparison of R3HDM4 methylation levels between tumor tissue samples and normal tissues. **(F)** Identification of tumor stage-specific methylation alterations. **(G)** Correlation between methylation levels of individual R3HDM4 CpG sites and CNV status (deep deletion, loss, neutral, gain, amplification). *P < 0.05, **P < 0.01, ***P < 0.001, ****P < 0.0001. CpG, Cytosine-phosphate-Guanine dinucleotide; KIRC, Kidney Renal Clear Cell Carcinoma; TCGA, The Cancer Genome Atlas; TNM, Tumor-Node-Metastasis staging system; CNV, copy number variation.

DNA methylation is pivotal in KIRC phenotypic changes and clinical outcomes, regulating tumor biology and patient prognosis. Given R3HDM4’s aberrant expression in KIRC, its epigenetic regulation was explored through DNA methylation analysis to identify overexpression mechanisms. R3HDM4’s promoter contains multiple CpG sites, with predominant hypermethylation in tumor tissues (circular/linear gene maps, **Figures 5A, B**). Tumor tissues had lower R3HDM4 methylation than adjacent normal tissues, correlating with its elevated tumor expression (**Figure 5C**). Correlation analysis showed weak yet significant inverse correlations between individual CpG sites (cg12045715; cg03052794) and aggregated methylation levels (**Figure 5D**). Contrary to **Figure 4C**, violin plots revealed significantly higher methylation beta-values in tumors vs. normal tissues for CpG sites cg02667291, cg25814612 et al.; in contrast, cg03052794 et al. and the aggregated region showed the inverse pattern (**Figure 5E**). Stage-stratified boxplots indicated differential methylation across stages for CpG sites cg02667291, cg12045715 and the aggregated region, with highest methylation in Stage 1 (**Figure 5F**). Integrative copy number variation (CNV) and methylation analysis revealed significant epigenetic-genomic interactions; various CNV states (deep deletion [−2], loss [−1], neutral [0], gain [1], amplification [2]) correlated with distinct methylation patterns. Notably, CpG sites cg02667291, cg25814612 et al. and the aggregated region had highest methylation in neutral (0) and amplification (2) CNV states, indicating CNV-dependent methylation variations (**Figure 5G**). Collectively, these findings identify R3HDM4 methylation as a molecular determinant in KIRC progression, with potential diagnostic and prognostic value for clinical management. 

### Functional analysis of R3HDM4 co-expression networks in KIRC

**Figure 6:**
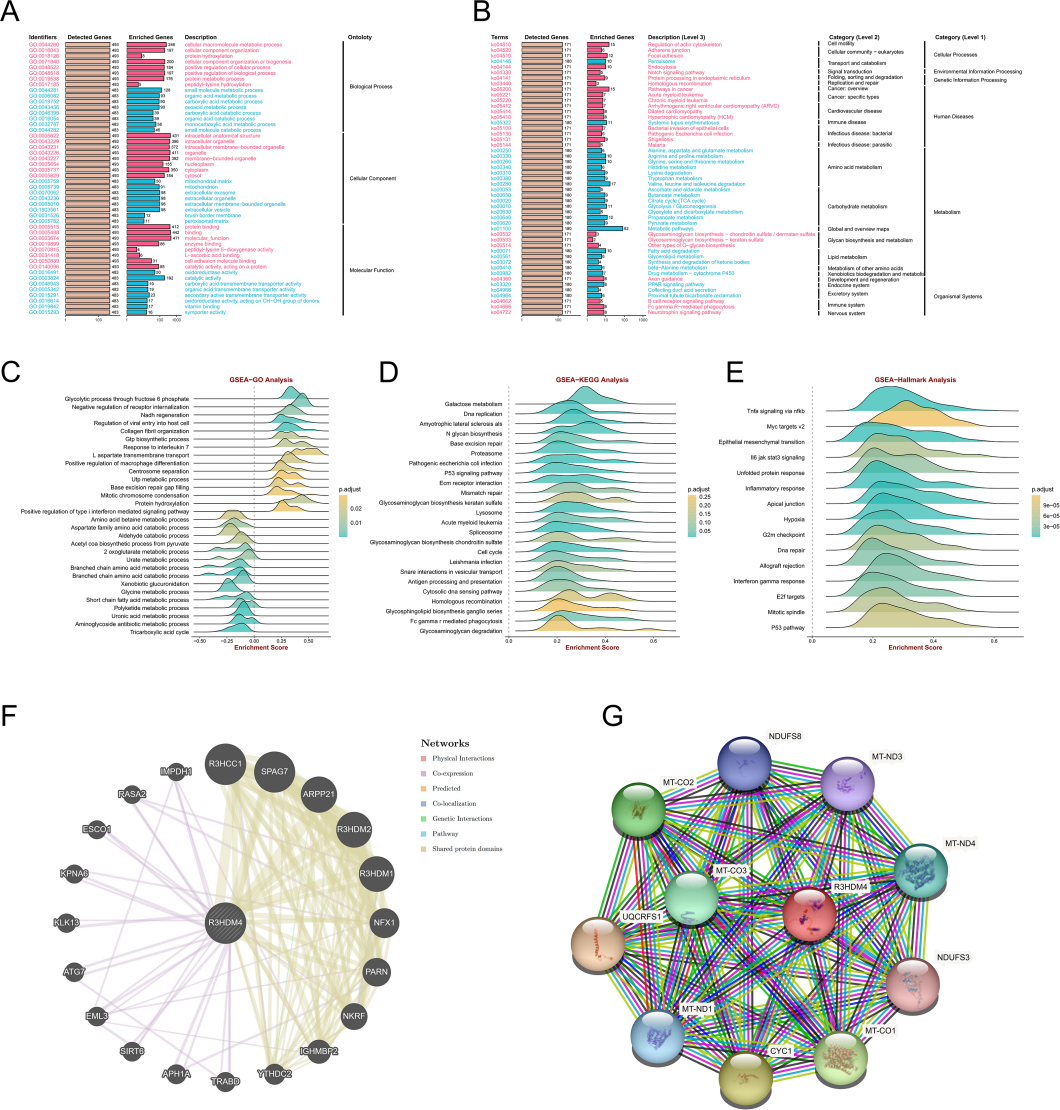
R3HDM4 functional enrichment analysis across immune-related pathways and biological processes in KIRC. **(A)** GO enrichment analysis of biological processes associated with R3HDM4. **(B)** KEGG pathway enrichment analysis of R3HDM4. **(C)** GSEA-GO enrichment profile of R3HDM4 in immune regulation, as indicated by the enrichment score. **(D)** GSEA-KEGG enrichment profile of R3HDM4 in immune regulation, as indicated by the enrichment score. **(E)** Hallmark gene set enrichment analysis of R3HDM4 in KIRC. **(F)** Protein–protein interaction network analysis of R3HDM4 in KIRC. **(G)** Gene co-expression network analysis correlated with R3HDM4 expression patterns in KIRC. *P < 0.05, **P < 0.01, ***P < 0.001, ****P < 0.0001. KIRC, Kidney Renal Clear Cell Carcinoma; R3HDM4, R3H domain containing 4; ES, Enrichment Score; GSEA, Gene Set Enrichment Analysis; GO, Gene Ontology; KEGG, Kyoto Encyclopedia of Genes and Genomes; R3HDM4, R3H domain containing 4. TCGA, The Cancer Genome Atlas.

To further elucidate R3HDM4’s functions in KIRC, GO, KEGG, and Hallmark enrichment analyses were performed using its related DEGs from TCGA-KIRC. GO analysis showed BP enrichment in cellular macromolecule/protein metabolism and organization (**Figure 6A**), CC in intracellular structures/organelles/nucleoplasm, and MF in protein/enzyme binding, supporting R3HDM4 as a nucleic acid-binding protein in PPIs. KEGG enrichment linked it to cellular processes (actin cytoskeleton, adherens junction), transport/catabolism (peroxisome, endocytosis), signal transduction (Notch pathway) (**Figure 6B**), metabolic pathways (amino acid, carbohydrate, lipid metabolism), and diseases (cancer, immune disorders). GO GSEA generated a waterfall plot highlighting positive (glycolysis, type I interferon signaling) and negative (receptor internalization inhibition) pathways (**Figure 6C**), indicating R3HDM4 enhances energy metabolism while suppressing certain regulators. KEGG GSEA emphasized glycosaminoglycan degradation, Fc gamma R-mediated phagocytosis, and homologous recombination (**Figure 6D**), contributing to ECM remodeling, immune phagocytosis, and DNA repair in KIRC. Hallmark GSEA highlighted TNFα/NF-κB, MYC/E2F targets, interferon gamma response, and EMT (**Figure 6E**), implicating R3HDM4 in cell cycle control and immune regulation. PPI networks identified R3HDM4 as a central node interacting with SPAG7, RHOH, PARP1 (**Figure 6F**), and a mitochondrial network with oxidative phosphorylation elements (UQCRFS1, CYC1, MT-CO1/2/3) (**Figure 6G**), emphasizing its role in mitochondrial function. In summary, enrichment analyses confirm R3HDM4 promotes KIRC progression via metabolic reprogramming, cellular adhesion, DNA repair, and immune-related pathways.

### Single-cell RNA sequencing profiling of R3HDM4 expression in KIRC

**Figure 7:**
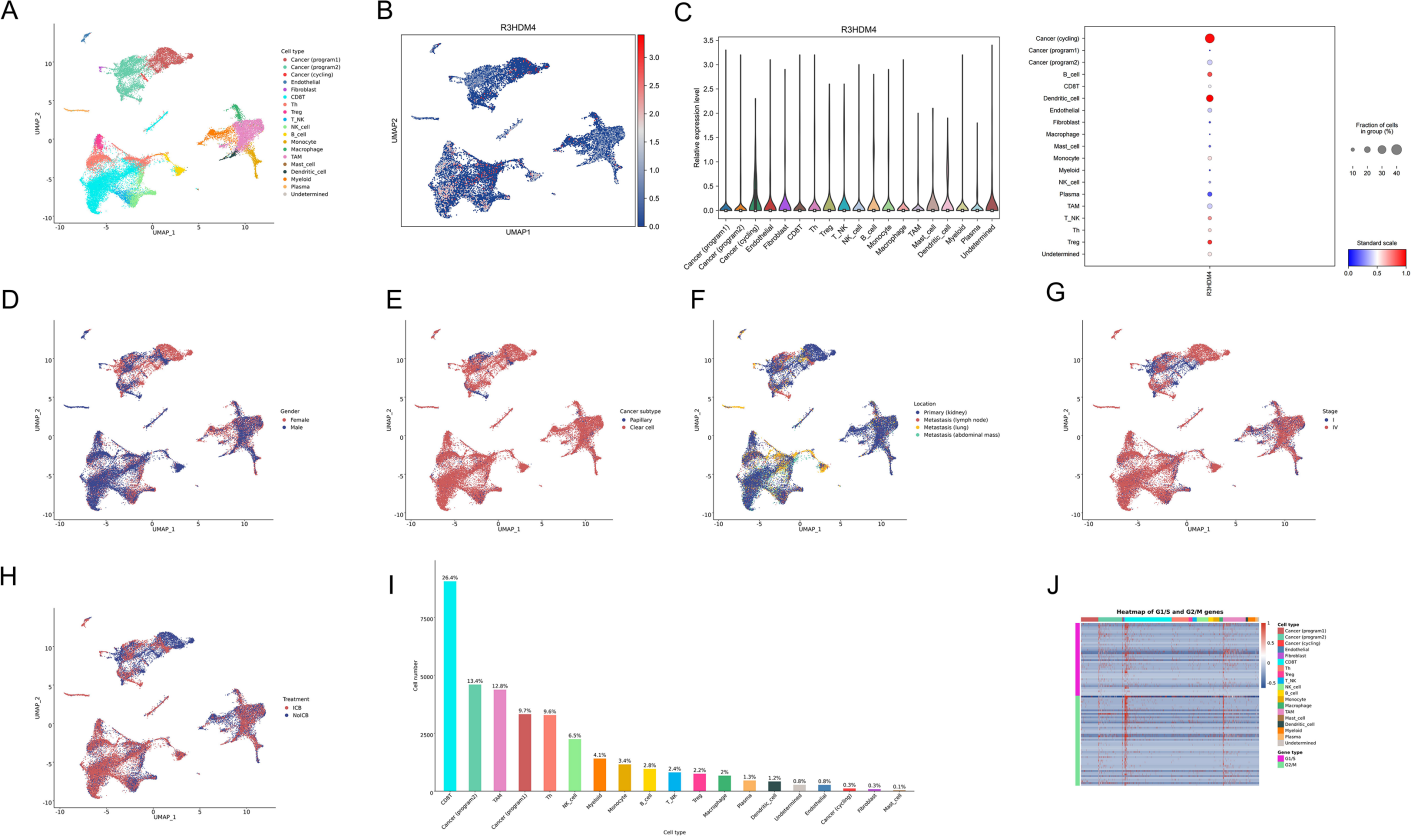
Single-cell RNA sequencing analysis of R3HDM4 expression in KIRC. **(A)** UMAP projection of scRNA-seq data colored by annotated cell types, including cancer (cycling/program1/program2), endothelial, fibroblast, mast cell, monocyte, myeloid, NK cell, plasma, dendritic cell, T cell, TAM, Treg, and undefined cells. **(B)** UMAP projection colored by R3HDM4 expression levels. **(C)** Bar plot of median normalized R3HDM4 expression across cell types. **(D)** UMAP projection stratified by gender. **(E)** UMAP projection stratified by cancer subtype. **(F)** UMAP projection stratified by sample location. **(G)** UMAP projection stratified by tumor stage. **(H)** UMAP projection stratified by treatment status (no UCB: blue, UCB: red). **(I)** Bar plot of cell type proportions in the dataset. **(J)** Heatmap depicting the expression of G1/S and G2/M cell cycle genes across annotated cell types. UMAP, Uniform Manifold Approximation and Projection; R3HDM4, R3H domain containing 4; CD4T_conv, Conventional CD4+ T cells; CD8T_typical, Typical CD8+ T cells; CD8T_exhausted, Exhausted CD8+ T cells; T_prolif, Proliferating T cells; Treg, Regulatory T cells; NK_cell, Natural Killer cell; B_cell, B lymphocyte; Mono/Macro, Monocyte/Macrophage; KIRC, Renal Clear Cell Carcinoma; CC, Cholangiocarcinoma; G1/S, G1/S phase transition genes; G2/M, G2/M phase transition genes.

To delineate R3HDM4 cellular distribution and expression in KIRC, single-cell RNA sequencing (scRNA-seq) data from KIRC tumors and adjacent normal tissues were analyzed. Uniform manifold approximation and projection (UMAP) dimensionality reduction identified distinct cell clusters, annotated via canonical marker genes into major lineages: cancer cells (cycling, program-specific subtypes), endothelial cells, fibroblasts, mast cells, monocytes, myeloid cells, natural killer (NK) cells, plasma cells, dendritic cells, regulatory T cells (Treg), tumor-associated macrophages (TAM), and undefined populations (**Figure 7A**). R3HDM4 expression on UMAP plots showed predominant upregulation in cancer cell clusters, with scattered expression in immune and stromal compartments (**Figure 7B**). Median normalized expression quantification across cell types confirmed highest R3HDM4 in cancer cells, followed by dendritic cells, TAM, myeloid cells, and minimal/absent expression in endothelial and plasma cells (**Figure 7C**). Clinical variable stratification contextualized R3HDM4’s role; gender-based analysis showed no significant clustering bias, with slight enrichment in male-derived cells (**Figure 7D**); clear cell populations predominantly occupied R3HDM4-high regions by subtype (**Figure 7E**); tumor location analysis distinguished primary kidney, lung metastases, and adjacent normal tissue, with metastatic cells showing higher R3HDM4 (**Figure 7F**); Stage IV tumors had higher R3HDM4 intensity than earlier stages (**Figure 7G**); R3HDM4 was enriched in ICB-treated cohorts, with distinct clustering in patients with/without urothelial carcinoma immunotherapy (UCI), suggesting therapeutic modulation of R3HDM4-expressing cells (**Figure 7H**). Cell type composition analysis identified CD8 T cells as the major sequenced population, followed by cancer cells, TAM, T cells, and other minor fractions, highlighting a tumor-dominant microenvironment (**Figure 7I**). A G1/S and G2/M phase gene heatmap across cell types demonstrated cell cycle activity primarily in cycling cancer subsets, correlating with R3HDM4 expression and implicating it in proliferative dynamics (**Figure 7J**). These scRNA-seq findings position R3HDM4 as a cancer-enriched factor in the KIRC TME, potentially regulating immune infiltration and metastatic potential.

### Correlations of R3HDM4 expression with immune cell infiltration in KIRC

**Figure 8:**
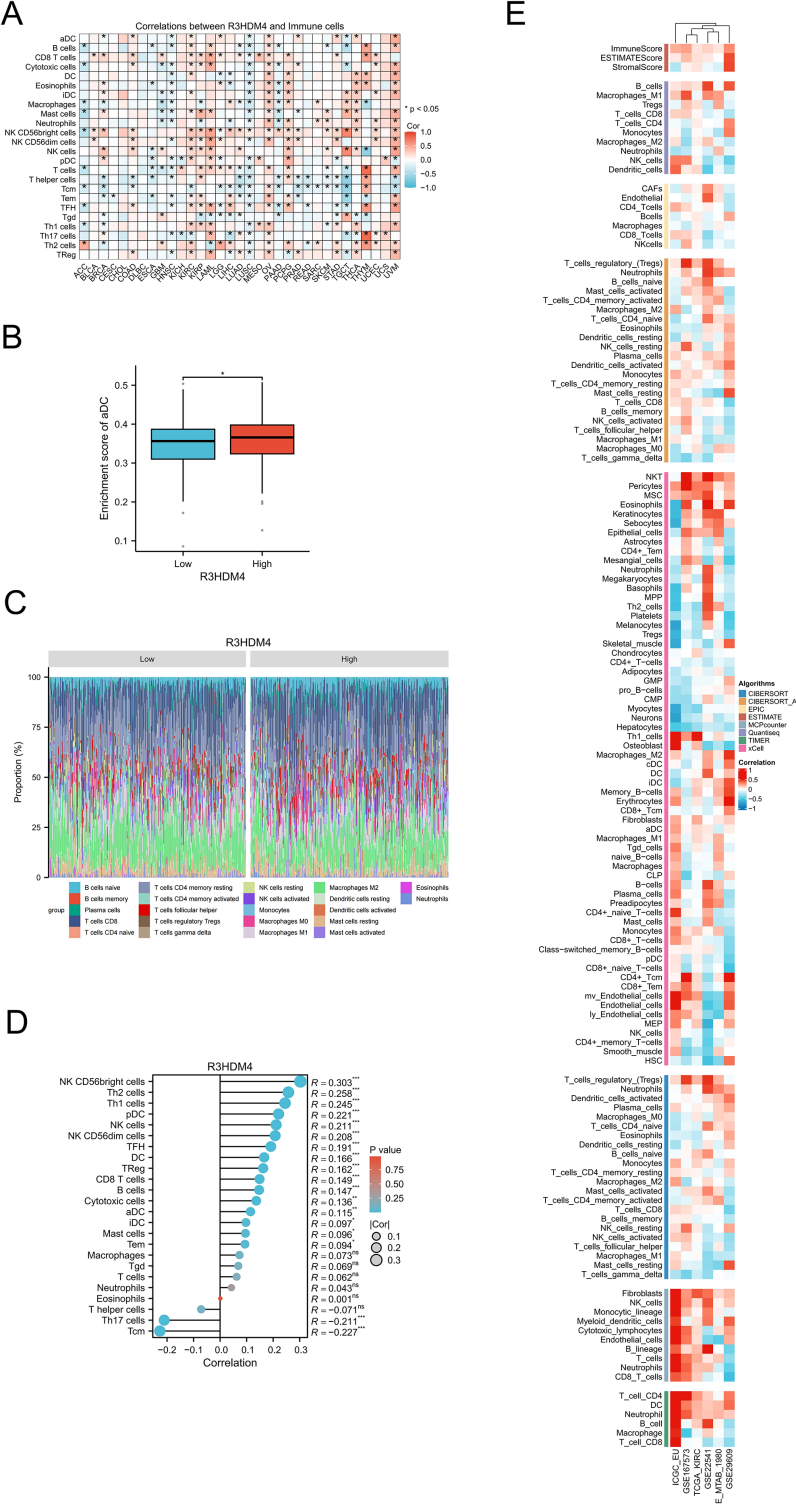
Integrative analysis of the correlation between R3HDM4 expression and tumor microenvironment immune cells in KIRC. **(A)** Correlation between R3HDM4 expression and immune cells across 33 cancer types. **(B)** Enrichment score of activated dendritic cells in patients with KIRC stratified by R3HDM4 expression (Low vs. High). **(C)** Comparison of immune cell proportions stratified by R3HDM4 expression levels (Low vs. High) in TCGA-KIRC. **(D)** Correlation between R3HDM4 expression and immune infiltration in KIRC using the ssGSEA algorithm. **(E)** Correlation between R3HDM4 expression and immune infiltration in KIRC across multiple immune infiltration tools and genomic datasets. *P < 0.05, **P < 0.01, ***P < 0.001, ****P < 0.0001. CIBERSORT, cell-type identification by estimating relative subsets of RNA Transcripts; Cor, Pearson correlation coefficient; ESTIMATE, estimation of stromal and immune cells in malignant tumor tissues using expression data; KIRC, Kidney Renal Clear Cell Carcinoma; Pval, p-value; TCGA, The Cancer Genome Atlas; xCell, cell type enrichment analysis tool.

In KIRC, R3HDM4 expression correlated significantly with clinicopathological characteristics, highlighting its potential role in tumor progression; tumor-infiltrating lymphocytes are strong indicators of tumor progression, histological differentiation, and nodal involvement, while complex crosstalk among malignant cells, stromal components, and immune populations in the tumor microenvironment contributes critically to pathogenesis. To investigate these relationships, comprehensive bioinformatics analyses using TCGA and GEO data assessed associations between R3HDM4 expression and immune infiltration in KIRC. Pan-cancer analysis revealed tissue-specific correlations between R3HDM4 and immune cell composition (**Figure 8A**); R3HDM4 showed variable (positive/negative) associations with immune infiltration across 33 malignancies, with consistent positive correlations in KIRC, LAML, ovarian cancer (OV), pheochromocytoma and paraganglioma (PCPG), uveal melanoma (UVM) — particularly with NK CD56bright cells. Comparative analysis showed significantly higher activated dendritic cell (aDC) enrichment scores in high-R3HDM4 tumors, suggesting a potential regulatory role in dendritic cell activation (**Figure 8B**). Immune cell distribution (**Figure 8C**) differed notably between R3HDM4 expression groups, with high-expression samples showing increased quiescent CD4+ memory T cells, activated NK cells, and M1 macrophages. Correlation analyses (**Figure 8D**) revealed positive associations between R3HDM4 and NK CD56bright cells, Th2 lymphocytes, follicular helper T cells, and negative correlations with central memory T cells (Tcm) and Th17 cells. Methodological validation was performed using six platforms (EPIC, ESTIMATE, TIMER, MCP-Counter, QuanTIseq, xCell) across six independent cohorts (TCGA-KIRC, ICGC_EU, GSE167573, GSE22541, E-MTAB-1980, GSE29609), and convergent results supported that R3HDM4 participates in renal carcinoma immune microenvironment remodeling, potentially influencing tumor immunogenicity and treatment response (**Figure 8E**). 

### Analysis of immune regulatory genes, TMB, MSI, and immune checkpoints related to R3HDM4 in KIRC

**Figure 9:**
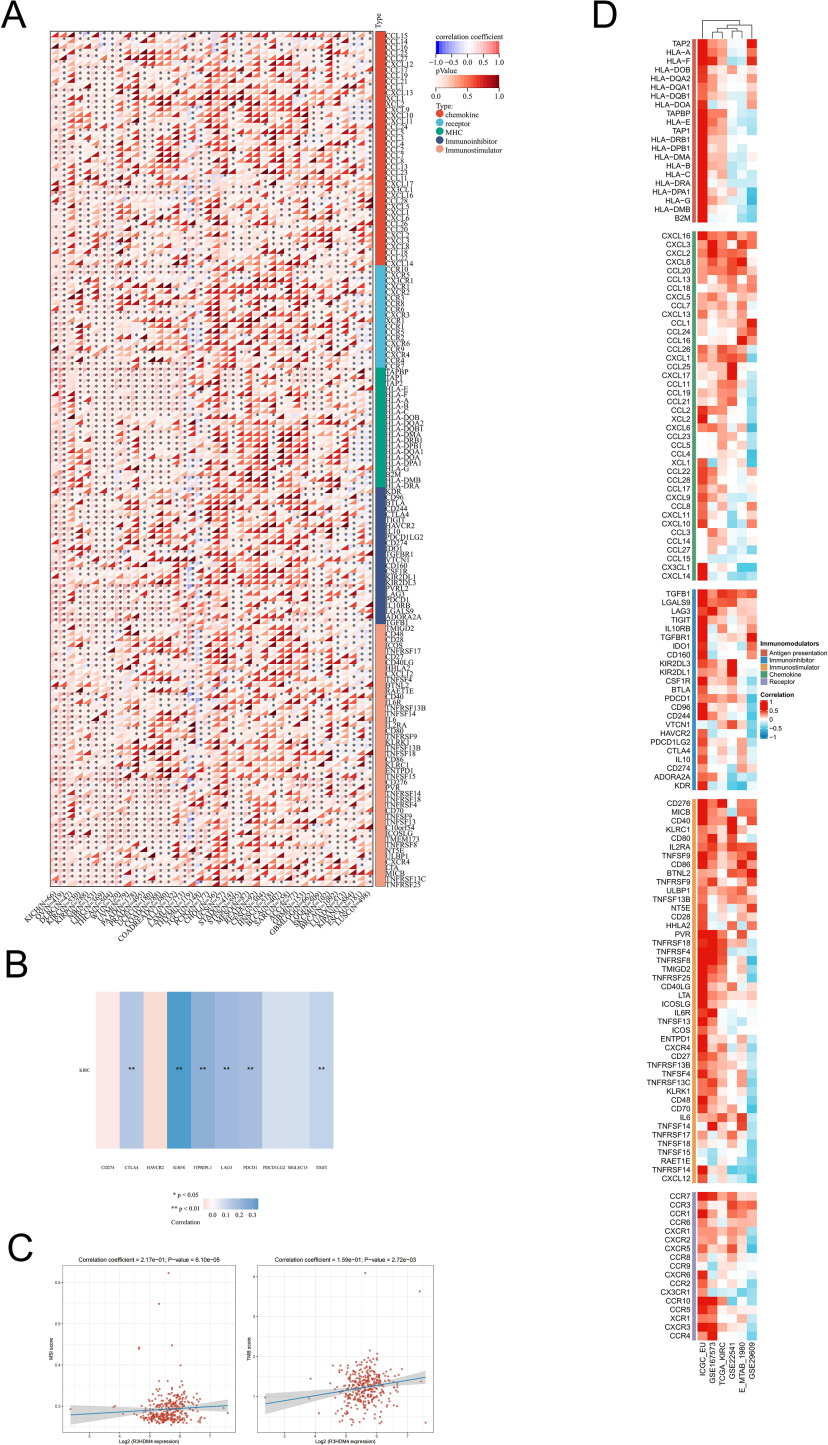
Integrated analysis of associations between R3HDM4 expression and immune-related genes. **(A)** Associations of R3HDM4 expression with TMB and MSI in KIRC. **(B)** Analysis of the relationship between R3HDM4 expression and immune checkpoints in KIRC. **(C)** Pan-cancer associations between R3HDM4 expression and immune-related genes. **(D)** Associations between R3HDM4 expression and immune-related genes in KIRC across multiple immune infiltration tools and genomic datasets. *P < 0.05, **P < 0.01, ***P < 0.001. KIRC, Kidney Renal Clear Cell Carcinoma; Pearson, Pearson correlation coefficient; Cor, Correlation coefficient.

To investigate R3HDM4’s role in KIRC immune-related processes, correlation analyses were conducted between R3HDM4 expression and immune-related genes, which identified significant associations with multiple immune gene categories in KIRC. Heatmap visualization showed strong positive correlations between R3HDM4 and chemokines (e.g., CCL15, CXCL12), immune checkpoint molecules (including PD-1, CTLA4), and major histocompatibility complex (MHC) genes (**Figure 9A**). Notably, R3HDM4 exhibited the strongest correlation with IGSF8, followed by ITPRIPL1, CD274 (PD-L1), CTLA4, LAG3, and TIGIT (**Figure 9B**). R3HDM4 expression also positively correlated with genomic instability markers (MSI, TMB), suggesting a potential link to tumor immunogenicity (**Figure 9C**). To validate these associations, comprehensive correlation analyses were performed between R3HDM4 and 137 immune modulators across five functional categories (antigen presentation molecules, chemokines, inhibitory checkpoints, stimulatory checkpoints, immune receptors) using six independent datasets (TCGA-KIRC, ICGC_EU, GSE167573, GSE22541, E-MTAB-1980, GSE29609) (**Figure 9D**). Consistent positive correlations with immunosuppressive checkpoints were observed, with TGFB1 showing the strongest association, followed by LGALS9 and LAG3. The consistent enrichment of these associations across multiple datasets reinforces findings reliability, indicating R3HDM4 significantly influences the immune landscape in the KIRC tumor microenvironment.

### R3HDM4 expression associates with immunotherapy response and drug sensitivity in KIRC

**Figure 10:**
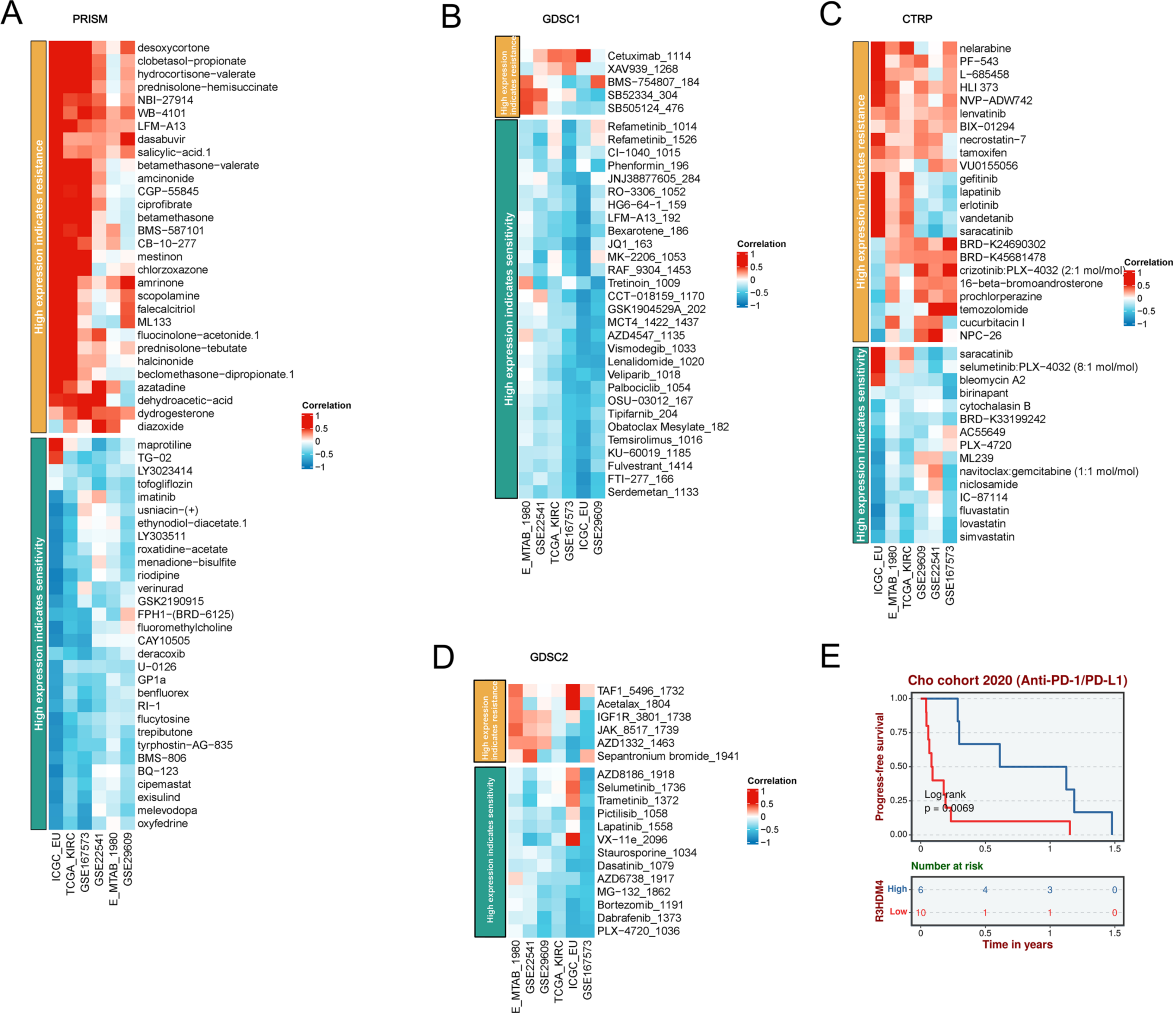
A comprehensive analysis of the correlation between R3HDM4 expression and drug response across multiple databases, along with its association with survival outcomes. **(A)** Correlation between R3HDM4 expression and drug resistance/sensitivity in the PRISM dataset. **(B)** Correlation between R3HDM4 expression and drug resistance/sensitivity in the GDSC1 database. **(C)** Correlation between R3HDM4 expression and drug resistance/sensitivity in the CTRP dataset. **(D)** Correlation between R3HDM4 expression and drug resistance/sensitivity in the GDSC2 dataset. **(E)** Overall survival analysis of the Cohort 2020 (Anti-PD-1/PD-L1). *P < 0.05, **P < 0.01, ***P < 0.001. CTRIP, Cancer Therapeutics Response Portal; PRISM, Preclinical Repurposing of Medicines; GDSC1/GDSC2, Genomics of Drug Sensitivity in Cancer 1/2; Anti-PD-L1, Anti-Programmed Death-Ligand 1; Log-rank, Log-rank test; Number at risk, Number of patients at risk at each time point.

To investigate R3HDM4 expression-drug sensitivity/resistance in KIRC, correlation analyses were performed using PRISM, GDSC1, CTRP, GDSC2 pharmacogenomic databases, integrating transcriptomic and drug response data from KIRC cell lines (**Figure 10**). In PRISM, higher R3HDM4 correlated positively with resistance to deoxycytidine-propanate, hydrocortisone-valerate, prednisone-hemisuccinate, and negatively with maprotiline, LY3023414, uracil-(+), ethynodiol-diacetate, indicating a dual role in chemotherapeutic response modulation (**Figure 10A**). In GDSC1, strong positive associations were observed with cisplatin, cetuximab, erlotinib resistance, and negative correlations with refametinib, CI-1040, tretinoin sensitivity, suggesting mechanisms involving DNA damage repair and EGFR signaling (**Figure 10B**). In CTRP/GDSC2, similar patterns emerged: positive correlations with TAF1-5496, Acetalax-1804, IGF1R-3801, JAK-8517 resistance, and negative correlations with AZD8186, Selumetinib, Trametinib sensitivity. Collectively, R3HDM4 may confer resistance to platinum-based/anti-EGFR therapies while enhancing sensitivity to MEK/ERK and proteasome inhibitors in KIRC (**Figures 10C, D**). Supporting these, Kaplan-Meier PFS analysis of Chao cohort (2020) KIRC patients treated with anti-PD-1/PD-L1 immunotherapy showed significantly longer median PFS (≈1 year extension) in high vs. low R3HDM4 expression patients (**Figure 10E**). These findings identify R3HDM4 as a potential biomarker for predicting ICB response and guiding personalized therapy in KIRC.

### Downregulation of R3HDM4 attenuated proliferation, migration, and invasion in KIRC cells

**Figure 11:**
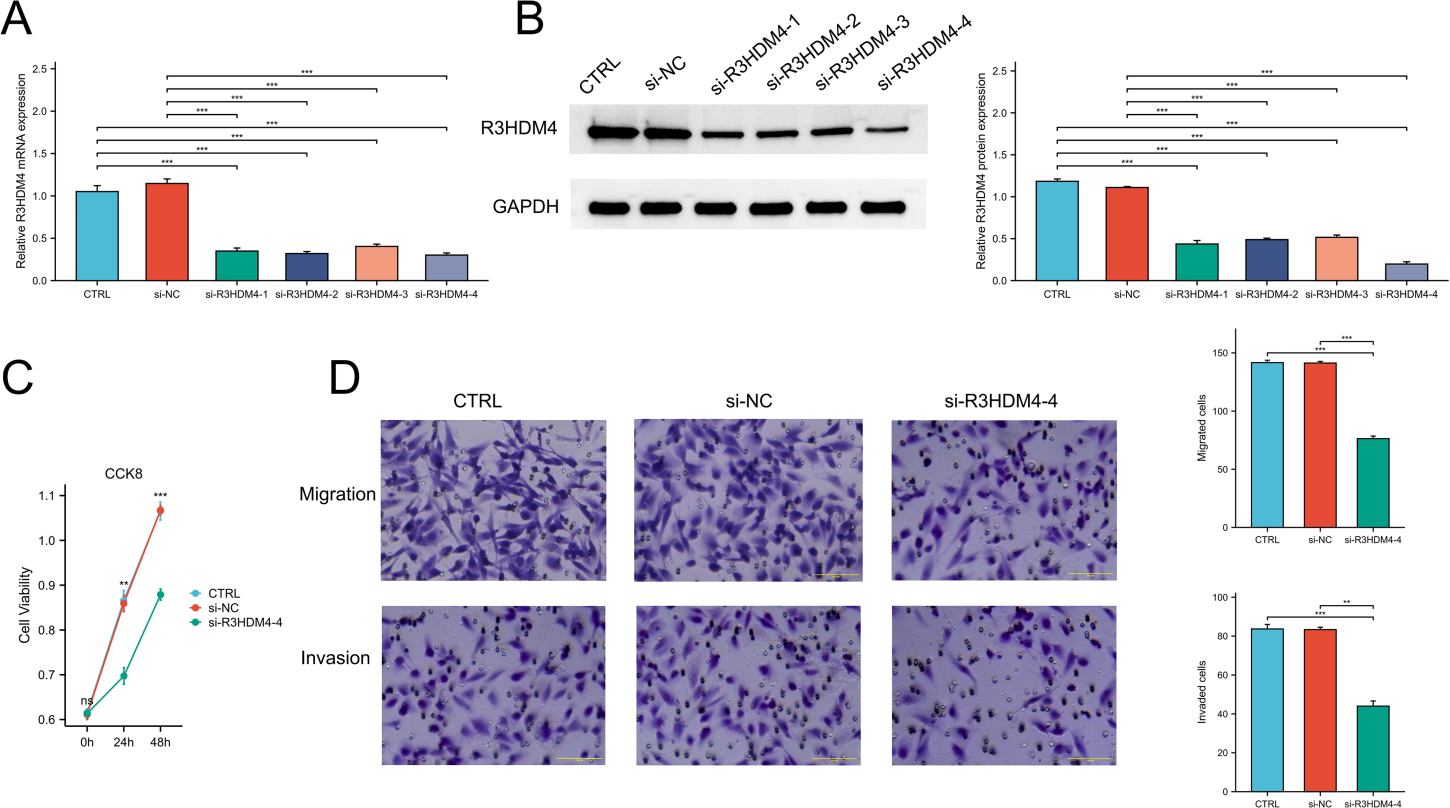
R3HDM4 silencing inhibits renal clear cell carcinoma cell proliferation, migration, and invasion in 786-O cells. **(A)** Validation of R3HDM4 mRNA knockdown efficiency in 786-O cells. **(B)** Validation of R3HDM4 protein knockdown efficiency in 786-O cells (representative Western blot included). **(C)** Cell viability of 786-O cells with R3HDM4 silencing assessed by CCK-8 assay. **(D)** Migration and invasion of 786-O cells with R3HDM4 silencing (representative images and quantification).*P < 0.05, **P < 0.01, ***P < 0.001. CTRL, control untreated; si-NC, negative control siRNA; si-R3HDM4, R3HDM4-targeting siRNA.

**Figure 12:**
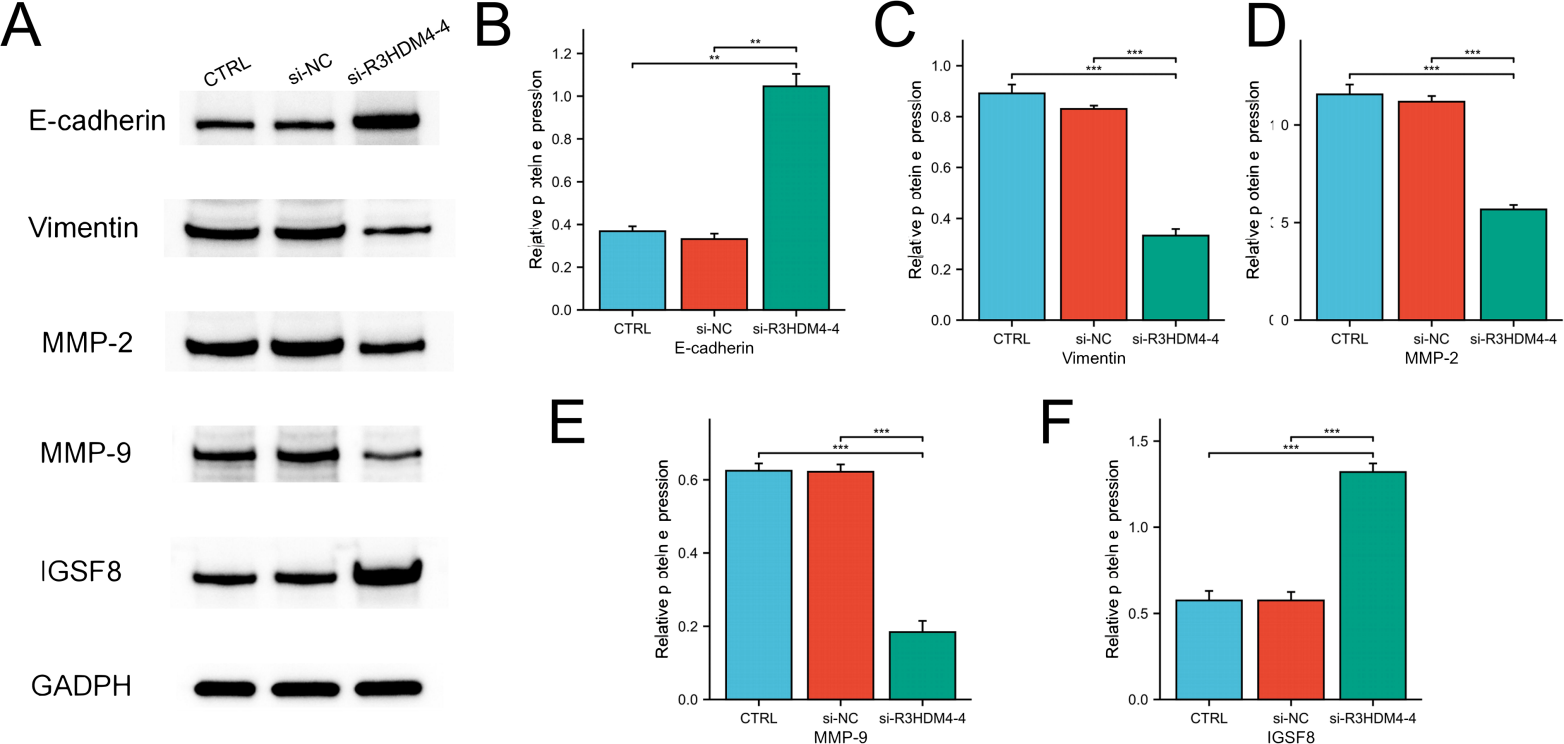
R3HDM4 silencing modulates expression of EMT, invasion, and immune-related markers in 786-O cells. **(A)** Representative Western blot of E-cadherin, MMP-2, MMP-9, Vimentin, IGSF8 and GAPDH in 786-O (control), 786-O+si-NC (negative control siRNA), and 786-O+si-R3HDM4-4 (R3HDM4-targeting siRNA) groups. **(B)** Quantification of relative E-cadherin expression across 786-O, si-NC, and si-R3HDM4–4 groups. **(C)** Quantification of relative MMP-2 expression across 786-O, si-NC, and si-R3HDM4–4 groups. **(D)** Quantification of relative MMP-9 expression across 786-O, si-NC, and si-R3HDM4–4 groups. **(E)** Quantification of relative Vimentin expression across 786-O, si-NC, and si-R3HDM4–4 groups. **(F)** Quantification of relative IGSF8 expression across 786-O, si-NC, and si-R3HDM4–4 groups.*P -*values were determined by one-way ANOVA; **P < 0.001 indicates statistical significance.*P < 0.05, **P < 0.01, ***P < 0.001. CTRL, control untreated; si-NC, negative control siRNA; si-R3HDM4, R3HDM4-targeting siRNA; E-cadherin, epithelial cadherin; MMP-2/9, matrix metalloproteinase-2/9; IGSF8, Immunoglobulin Superfamily Member 8 expression.

To elucidate R3HDM4’s biological function in KIRC progression, *in vitro* studies were performed in R3HDM4-knockdown 768-O cells. Four R3HDM4-targeting siRNAs were transfected, and effective R3HDM4 silencing was verified by Western blot and quantitative PCR, with significant differences observed versus untreated and si-NC-treated controls (**Figures 11A, B**). Following comprehensive assessment, the most effective si-R3HDM4–4 was selected for subsequent functional assays. CCK-8 assay showed R3HDM4 depletion significantly impaired 768-O cell proliferation (**Figure 11C**), while transwell assays demonstrated R3HDM4 silencing notably reduced cancer cell migration and invasion (**Figure 11D**). Given EMT’s established role in cancer metastasis, key EMT markers were analyzed; EMT is characterized by decreased epithelial markers (e.g., E-cadherin), increased mesenchymal markers (e.g., vimentin) and matrix metalloproteinases (MMP-2, MMP-9), which collectively enhance cellular motility and invasiveness *in vitro (*37–39). Western blot analysis revealed R3HDM4 knockdown significantly upregulated E-cadherin and downregulated vimentin, MMP-2, and MMP-9 (**Figures 12A-E**). These findings confirm R3HDM4 promotes KIRC progression by regulating EMT-related signaling pathways that control neoplastic cell proliferation and metastatic potential .

### R3HDM4 silencing upregulated IGSF8 protein expression in KIRC cells

This study further systematically examined the associations between R3HDM4 expression patterns and immune cell infiltration characteristics in the tumor microenvironment. Analyses identified significant positive correlations between R3HDM4 and key immunosuppressive checkpoint molecules, with a strong positive correlation between R3HDM4 and IGSF8. To validate this functional association, siRNA-mediated R3HDM4 knockdown was performed in 768-O cells; Western blot (**Figures 12A, F**) showed R3HDM4 silencing (si-R3HDM4-4) significantly upregulated IGSF8 protein vs. CTRL and si-NC groups. Densitometric quantification confirmed a negative regulatory relationship between R3HDM4 and IGSF8 at the protein level, which contrasts with the strong transcriptomic positive correlation from prior bioinformatic analysis, suggesting a potential post-transcriptional or post-translational regulatory mechanism.

## Discussion

KIRC is the most common renal malignancy subtype. While VEGF-targeted TKIs and ICIs significantly improve advanced patient survival, key challenges (immunotherapy resistance, intratumoral heterogeneity, lack of reliable biomarkers) still limit long-term efficacy ) (2, 3, 13). This highlights the urgent need for novel molecular drivers linking tumor cell-intrinsic behaviors (proliferation, metastasis) with TME remodeling, as such targets serve as biomarkers and therapeutic vulnerabilities. R3HDM4, encoding an R3H domain-containing RNA metabolism protein, is poorly characterized in cancers with no prior KIRC studies (20, 21). Prompted by this, we investigated R3HDM4’s expression, function, and clinical significance in KIRC using multi-omics datasets, *in vitro* experiments, and clinical correlation analyses. Pan-cancer analysis showed R3HDM4 upregulation in solid tumors (including KIRC) vs. normal tissues, with downregulation only in DLBC, mirroring tissue-specific dysregulation of cancer drivers and suggesting oncogenic potential. In KIRC, consistent R3HDM4 upregulation across datasets correlated with advanced stages and higher grades, similar to known drivers BUB1B and EMX2 *in vitro* (16, 17). R3HDM4’s association with KIRC aggressiveness extends beyond BUB1B (cell cycle-related) and EMX2 (tissue-specific), linking to both stage and grade. Renal cancer cell lines showed variable R3HDM4 expression (high in 786-O; low in KMRC-1), reflecting KIRC molecular diversity and supporting its potential as a subtype-specific malignancy marker. 

To validate bioinformatics findings, *in vitro* experiments confirmed higher R3HDM4 protein levels in KIRC via IHC and qRT-PCR/Western blot (786-O, HEK-293T cells). This multi-method approach addressed early cancer gene research limitations of relying solely on transcriptomic data, as post-transcriptional regulation causes mRNA-protein discrepancies that limited clinical utility of previous KIRC biomarkers. R3HDM4 was a clinically relevant prognostic marker: pan-cancer Cox regression linked its high expression to poor OS in KIRC, LAML, LGG; Kaplan-Meier analysis and time-dependent ROC supported its KIRC prognostic value. Multivariate analysis confirmed it as an independent prognostic factor unaffected by stage/grade, with external validation in independent cohorts ensuring reliability and resolving insufficient validation in prior KIRC biomarker studies *in vitro*.

Weak inverse overall R3HDM4 methylation-expression correlation was observed in analyzing epigenetic mechanisms of its KIRC upregulation via DNA methylation. Site-specific analysis revealed varied CpG methylation patterns, with some hypermethylated and others hypomethylated; CNVs correlated with distinct methylation patterns, suggesting genomic-epigenetic interaction. These findings align with prior KIRC epigenetic research (exemplified by VHL) showing gene expression regulated by multiple loci and genomic features. Our study highlights R3HDM4 expression controlled by complex methylation-CNV interactions, providing new insights into KIRC gene regulation. 

Functional enrichment analyses linked R3HDM4 to metabolic reprogramming, EMT, and immune pathways in KIRC progression. R3HDM4 was an upstream regulator of metabolic reprogramming, differing from previous studies focusing on downstream enzymes; its EMT association supported *in vitro* findings, while PPI networks showed interactions with mitochondrial/DNA repair proteins, implying roles in therapy resistance and metabolic adaptation. This highlighted R3HDM4’s broad regulatory influence, distinct from specific drivers like BUB1B.

scRNA-seq revealed R3HDM4’s predominant expression in cancer cell clusters, especially cycling/metastatic subsets, with low expression in immune/stromal cells; unlike bulk RNA-seq averaging signals, scRNA-seq pinpointed its cancer cell-specific pro-tumor effects and indirect TME influence. Elevated R3HDM4 was detected in metastatic cells, Stage IV tumors, and ICI-treated cohorts, linking it to aggressive KIRC phenotypes and potential immunotherapy response. These findings extend prior scRNA-seq studies by identifying cancer cell-enriched genes with prognostic and therapeutic implications.

R3HDM4 expression correlated context-dependently with immune cell infiltration: positive with NK CD56bright cells, M1 macrophages et al. and negative with Tcm, Th17 cells. These align with NK/M1 anti-tumor roles, while Tcm (long-term immune memory) negative correlation explains R3HDM4’s poor prognostic link despite anti-tumor subset associations. Validated across six platforms (EPIC et al.) and six cohorts, the results distinguish our study from single-platform/small-cohort studies, indicating R3HDM4 regulates KIRC TME—seldom reported for RNA metabolism-related genes. R3HDM4’s immunomodulatory role was clarified via correlations with immune regulatory genes (CCL15, PD-1 et al.), MHC genes, IGSF8 (immune adhesion/tumor immunity), TMB, and MSI (genomic instability/ICI response markers). Consistent correlations position it as a potential TMB/MSI surrogate (9, 11). KM analysis of Chao (2020) cohort showed elevated R3HDM4 associated with improved PFS in ICI-treated KIRC patients, supporting its utility as a complementary predictive biomarker for immunotherapy patient stratification. 

Pharmacogenomic analyses demonstrated R3HDM4 expression correlates with drug sensitivity: positive correlations with platinum-based (cisplatin) and anti-EGFR (cetuximab) therapy resistance, and negative correlations with MEK/ERK inhibitor (refametinib, selumetinib) sensitivity. These results align with established platinum resistance mechanisms and MEK/ERK pathway activation in KIRC. R3HDM4 was identified as a potential treatment selection biomarker, addressing the limitation of lacking biomarkers for MEK inhibitor-responsive KIRC patients; our data suggest its expression enables personalized MEK inhibitor application in high R3HDM4-expressing patients. 

*In vitro* assays confirmed R3HDM4’s pro-tumorigenic role. siRNA knockdown of R3HDM4 in 768-O cells significantly reduced proliferation and migratory/invasive capacities, with EMT phenotype reversal marked by E-cadherin upregulation and vimentin et al. downregulation. This supports the EMT-KIRC metastasis link and identifies R3HDM4 as a novel upstream pathway regulator. Unlike traditional EMT drivers (Snail, Twist) that directly repress E-cadherin transcription, R3HDM4’s RNA metabolism role suggests post-transcriptional EMT regulation, potentially via EMT marker mRNA stability modulation. This novel KIRC pathway merits further investigation. 

Notably, R3HDM4 and IGSF8 exhibit regulatory interaction. Transcriptomic data showed strong positive correlation between them, yet R3HDM4 knockdown significantly upregulated IGSF8 protein expression, indicating inverse post-transcriptional/post-translational regulation. This discrepancy supports R3HDM4’s RNA metabolism function, leading to hypotheses such as repressed IGSF8 mRNA translation or facilitated degradation via R3HDM4-mediated microRNA/RNA-binding protein interactions, (19, 20). No prior studies reported R3HDM4-IGSF8 functional association in cancer, highlighting this novel regulatory axis that may influence KIRC immunomodulatory phenotypes. 

In conclusion, this study identified R3HDM4 as a novel oncogenic driver in KIRC, with roles including promoting tumor proliferation and metastasis through EMT regulation, modulating TME composition and immune checkpoint expression, correlating with TMB/MSI and ICI response, and predicting sensitivity to MEK/ERK inhibitors. These findings fill significant gaps in the current understanding of R3HDM4's function in cancer and KIRC biology, while highlighting its potential as a prognostic biomarker independent of cancer stage and as a predictive marker for personalized therapy.

This study's limitations include using only one KIRC cell line (786-O) and normal renal cells (HEK-293T) *in vitro*, highlighting the need for *in vivo* validation with patient-derived xenografts and other KIRC models. The small IHC cohort suggests the need for larger multi-center studies to confirm clinical correlations. Further investigation is needed into the molecular mechanisms of R3HDM4's regulation of IGSF8 and EMT, especially post-transcriptional pathways. Despite these limitations, R3HDM4 shows promise for clinical translation, potentially addressing therapy resistance and improving personalized treatments for KIRC patients.

## Data Availability

The data presented in the study are retrieved from public repositories, including the TCGA repository (https://portal.gdc.cancer.gov/, accession: TCGA-KIRC), GTEx repository (http://gtexportal.org/), GEO repository (https://www.ncbi.nlm.nih.gov/geo/, accessions: GSE167573, GSE22541 and GSE29609), the ICGC portal (https://dcc.icgc.org/) and ArrayExpress repository (https://www.ebi.ac.uk/arrayexpress/),accession: E_TABM_1980).

## Glossary

R3HDM4: R3H domain containing 4
KIRC: Kidney Renal Clear Cell Carcinoma
AFP: Alpha-fetoprotein
TCGA: The Cancer Genome Atlas
GEO: Gene Expression Omnibus
ICGC: International Cancer Genome Consortium
IHC: immunohistochemistry
AUC: Area Under Curve
CI: Confidence Interval
ROC: Receiver Operating Characteristic
TPM: Transcripts Per Million
CC: Cholangiocarcinoma
CNV: Copy Number Variation
SNV: Single Nucleotide Variant
WES: Whole Exome Sequencing
CpG: Cytosine-phosphate-Guanine dinucleotide
KIRC: Renal Clear Cell Carcinoma
TNM: Tumor-Node-Metastasis staging system
ES: Enrichment Score
GSEA: Gene Set Enrichment Analysis
CIBERSORT: cell-type identification by estimating relative subsets of RNA Transcripts
Cor: Pearson correlation coefficient
ESTIMATE: estimation of stromal and immune cells in malignant tumor tissues using expression data
xCell: cell type enrichment analysis tool
Pearson: Pearson correlation coefficient
Cor: Correlation coefficient
UMAP: Uniform Manifold Approximation and Projection
CD4T_conv: Conventional CD4+ T cells
CD8T_typical: Typical CD8+ T cells
CD8T_exhausted: Exhausted CD8+ T cells
T_prolif: Proliferating T cells
Treg: Regulatory T cells
NK_cell: Natural Killer cell
B_cell: B lymphocyte
Mono/Macro: Monocyte/Macrophage
KIRC: Renal Clear Cell Carcinoma
CC: Cholangiocarcinoma
G1/S: G1/S phase transition genes
G2/M: G2/M phase transition genes
CTRP: Cancer Therapeutics Response Portal
PRISM: Preclinical Repurposing of Medicines
GDSC1/GDSC2: Genomics of Drug Sensitivity in Cancer 1/2
Anti-PD-1: Anti-Programmed Cell Death Protein 1
Anti-CTLA-4: Anti-Cytotoxic T-Lymphocyte-Associated Protein 4
Log-rank: Log-rank test
Number at risk: Number of patients at risk at each time point
CTRL: control untreated
si-NC: negative control siRNA
si-R3HDM4: R3HDM4-targeting siRNA
E-cadherin: epithelial cadherin
MMP-2/9: matrix metalloproteinase-2/9
LUAD: Lung adenocarcinoma
LUSC: Lung squamous cell carcinoma
TMB: Tumor mutation burden;MSI, Microsatellite instability
GTEx: Genotype-Tissue Expression databases
OS: overall survival
PFS: progression-free survival
DSS: disease-specific survival
ROC: receiver operating characteristic
AUCs: area under the curves
C-index: consistency index
KM plotter: Kaplan-Meier plotter
IOD: integrated optical density
FC: fold-change
HR: Hazard ratio
siRNA: small interfering RNA
OD: optical density
GBM: Glioblastoma
GBMLGG: Glioblastoma and Lower Grade Glioma
LGG: Lower Grade Glioma
BRCA: Breast Cancer
KIRP: Kidney Papillary Cell Carcinoma
STAD: Stomach Adenocarcinoma
HNSC: Head and Neck Squamous Cell Carcinoma
KIRC: Kidney Renal Clear Cell Carcinoma
PAAD: Pancreatic Adenocarcinoma
TILs: tumor-infiltrating lymphocytes
TME: tumor microenvironment
ICI: Immune checkpoint inhibitors.
