# Supplementary figures and images for: R3HDM4 influences kidney renal clear cell carcinoma progression, immune modulation, and potential links to the IGSF8 immune checkpoint
Source: Front Immunol. 2025 Nov 19;16:1722358. doi: 10.3389/fimmu.2025.1722358 (PMC12672864; doi:10.3389/fimmu.2025.1722358)

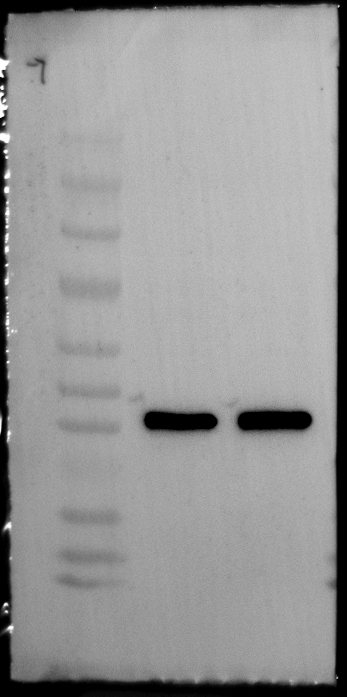

Supplement: Supplementary file 1 [file DataSheet1.zip › Fig3D-GAPDH.jpg]

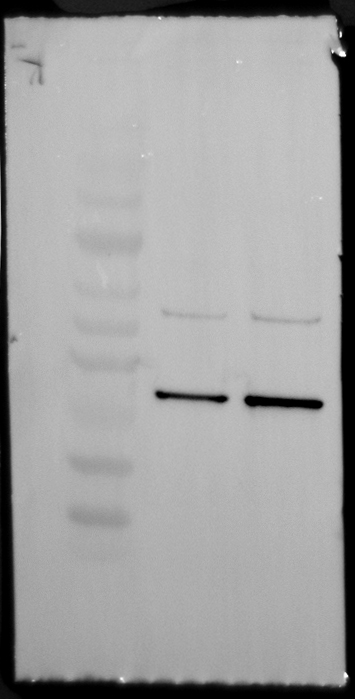

Supplement: Supplementary file 1 [file DataSheet1.zip › Fig3D-R3HDM4.jpg]

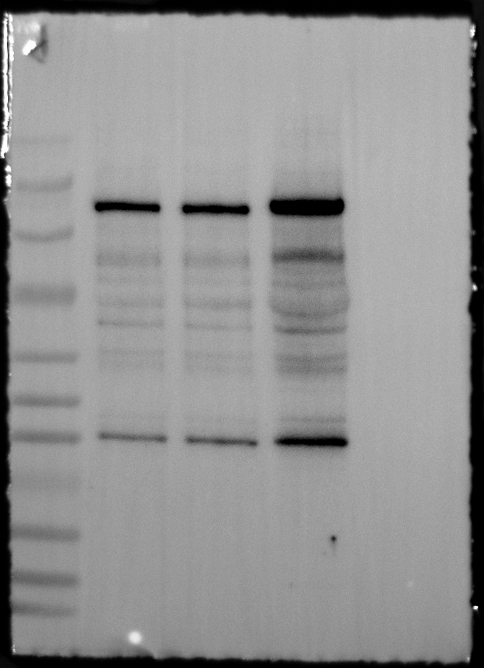

Supplement: Supplementary file 1 [file DataSheet1.zip › Fig12A-E-CADHERIN.jpg]

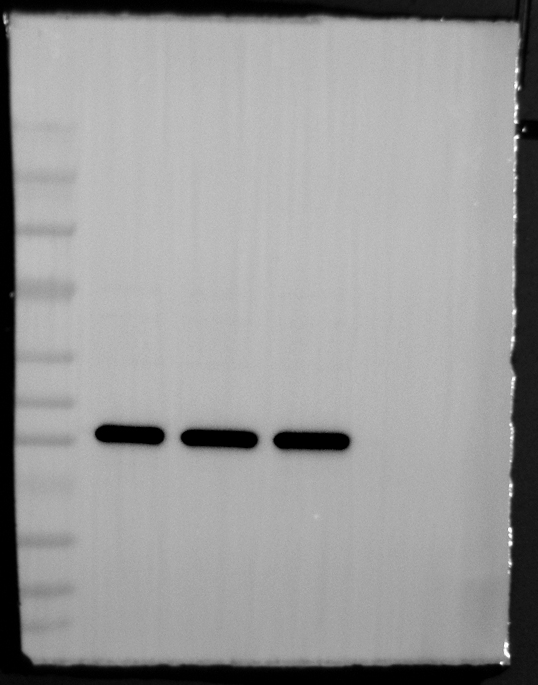

Supplement: Supplementary file 1 [file DataSheet1.zip › Fig12A-GAPDH.jpg]

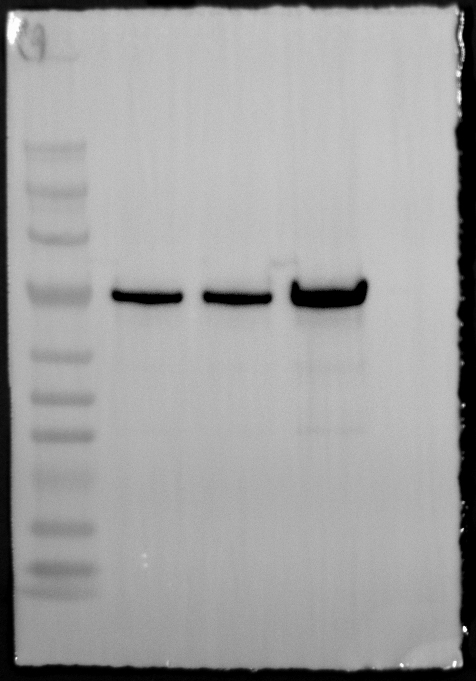

Supplement: Supplementary file 1 [file DataSheet1.zip › Fig12A-IGSF8.jpg]

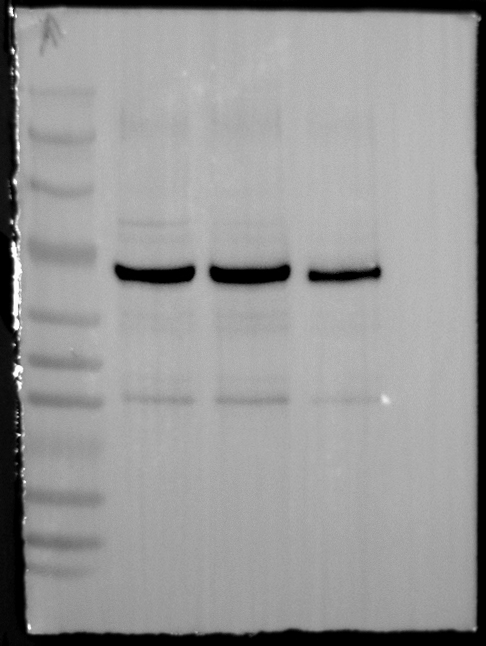

Supplement: Supplementary file 1 [file DataSheet1.zip › Fig12A-MMP2.jpg]

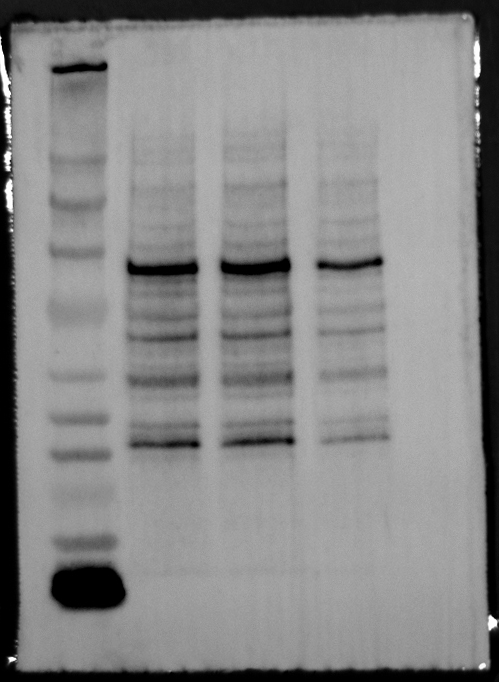

Supplement: Supplementary file 1 [file DataSheet1.zip › Fig12A-MMP9.jpg]

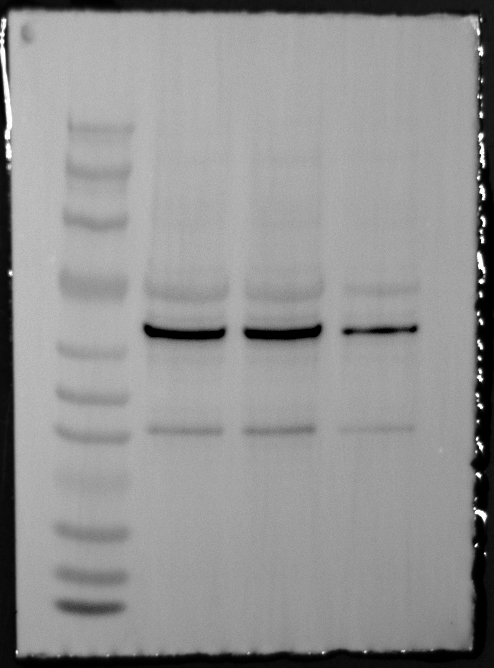

Supplement: Supplementary file 1 [file DataSheet1.zip › Fig12A-vimentin-2.jpg]

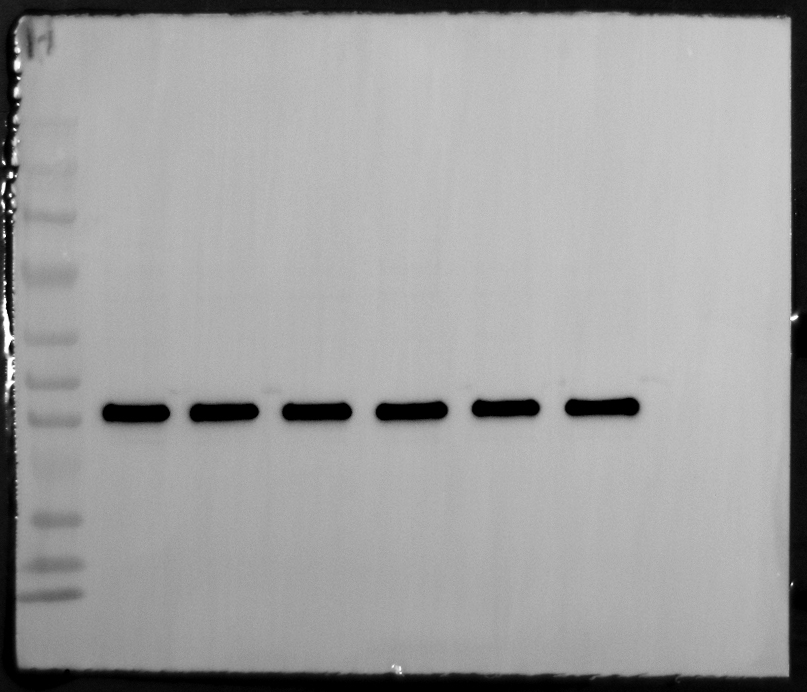

Supplement: Supplementary file 1 [file DataSheet1.zip › Fig11B-GAPDH.jpg]

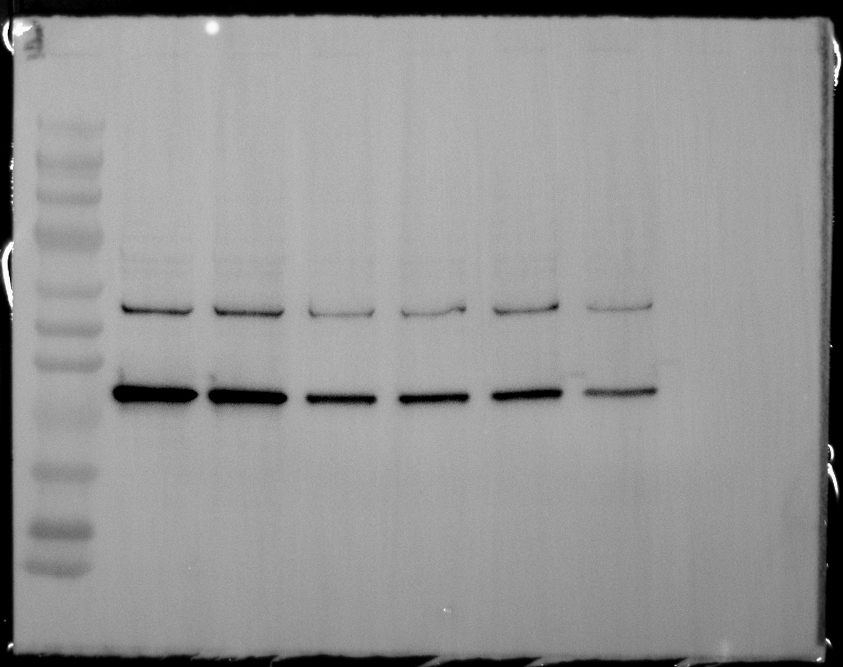

Supplement: Supplementary file 1 [file DataSheet1.zip › Fig11B-R3HDM4.jpg]
