# Supplementary material for: R3HDM4 influences kidney renal clear cell carcinoma progression, immune modulation, and potential links to the IGSF8 immune checkpoint
Source: Front Immunol. 2025 Nov 19;16:1722358. doi: 10.3389/fimmu.2025.1722358 (PMC12672864; doi:10.3389/fimmu.2025.1722358)
Supplement: Supplementary file 2 [file Table1.docx]

Table S1 Clinical characteristics of patients with TCGA-KIRC

| Characteristics | overall |
| --- | --- |
| Age, n (%) |  |
| <= 60 | 269 (49.7%) |
| > 60 | 272 (50.3%) |
| Gender, n (%) |  |
| Male | 354 (65.4%) |
| Female | 187 (34.6%) |
| Histologic grade, n (%) |  |
| G1&G2 | 250 (46.9%) |
| G3&G4 | 283 (53.1%) |
| Pathologic T stage, n (%) |  |
| T1&T2 | 350 (64.7%) |
| T3&T4 | 191 (35.3%) |
| Pathologic N stage, n (%) |  |
| N0 | 242 (93.8%) |
| N1 | 16 (6.2%) |
| Pathologic M stage, n (%) |  |
| M0 | 429 (84.4%) |
| M1 | 79 (15.6%) |
