# Supplementary material for: R3HDM4 influences kidney renal clear cell carcinoma progression, immune modulation, and potential links to the IGSF8 immune checkpoint
Source: Front Immunol. 2025 Nov 19;16:1722358. doi: 10.3389/fimmu.2025.1722358 (PMC12672864; doi:10.3389/fimmu.2025.1722358)
Supplement: Supplementary file 3 [file Table2.docx]

**Table S2**

| Characteristic | No.of patients(%) |
| --- | --- |
| **n** | **16** |
| **Age, n (%)** |  |
| ≤55 | 8(50.0%) |
| >55 | 8 (50.0%) |
| **Gender, n (%)** |  |
| Male | 9(56.3%) |
| Female | 7 (43.7%) |
| **Tumor size, n (%)** |  |
| ≦5cml | 6(37.5%) |
| >5cm | 10(62.5%) |
| **Differentiation, n (%)** |  |
| well | 0(0.0%) |
| Moderate | 12(75.0%) |
| Poor | 4 (25.0%) |
| **Lymph node metastasis, n (%)** |  |
| No | 16(100.0%) |
| Yes | 0 (0.0%) |
| **TNM stage, n (%)** |  |
| I-II | 12(75.0%) |
| III-IV | 4 (25.0%) |
